# Predictive Factors for Acute and Late Genitourinary and Gastrointestinal Toxicity Following Modern Radiotherapy for Prostate Cancer: A Narrative Review of Clinical, Dosimetric, Immunological, and Genetic Determinants

**DOI:** 10.3390/life16091477

**Published:** 2026-09-03

**Authors:** Rares-Nicolae Vadana, Adrian-Cornel Maier, Laurențiu Drăguș, Ștefan Roșca, Simona-Dana Mitincu-Caramfil, Roxana-Andreea Rahnea-Nita, Mihaela Dumitru, Raluca Barzu, Daniela Mihalcia, Laura-Florentina Rebegea

**Affiliations:** 1 Faculty of Medicine and Pharmacy, Research Centre in the Medical-Pharmaceutical Field, “Dunarea de Jos” University of Galati, 800008 Galati, Romania; 2 M Hospital, Str. Witting 10, 010903 București, Romania; 3 The Clinical Department, The Faculty of Medicine, “Carol Davila” University of Medicine and Pharmacy, 020021 Bucharest, Romania; 4 Radiotherapy Department, “Sf. Apostol Andrei” County Emergency Clinical Hospital, 800578 Galati, Romania; 5 Live Wins Association, 030923 Bucharest, Romania

**Keywords:** prostate cancer, radiotherapy, GU toxicity, GI toxicity, radiogenomics

## Abstract

Background: Despite advances in prostate cancer (PC) radiotherapy, including intensity-modulated radiotherapy (IMRT), volumetric modulated arc therapy (VMAT), and stereotactic body radiotherapy (SBRT), genitourinary (GU) and gastrointestinal (GI) toxicities remain important complications. Identifying reliable predictors is essential for personalized treatment. Objective: The aim of this study was to summarize current evidence on clinical, dosimetric, immunological, and genetic predictors of acute and late GU and GI toxicities after PC radiotherapy. Materials and Methods: A structured literature review of studies published between 2000 and 2026 was conducted using PubMed, Scopus, and Europe PMC. Eligible studies included clinical investigations, randomized controlled trials, meta-analyses, and reviews assessing toxicity according to RTOG and/or CTCAE criteria. Results: GU toxicity was associated with pretreatment International Prostate Symptom Score (IPSS), large prostate volume, prior TURP, smoking, alpha-blocker use, and bladder/urethral dose. GI toxicity correlated with rectal dose–volume parameters, anorectal irradiation, anticoagulant therapy, and cardiovascular comorbidities. Acute grade ≥ 2 toxicity predicts late toxicity. Preliminary data from small single-center cohorts associate elevated IL-6, TGF-β1, TNF-α, and systemic immune-inflammation index with higher toxicity risk, and higher lymphocyte counts with lower risk; these findings require prospective validation. The PROSTOX radiogenomic signature is promising for the prediction of late GU toxicity but still requires independent external validation in larger, ethnically diverse cohorts with longer follow-up, and is not currently suitable for routine clinical decision-making. Conclusions: GU and GI toxicities have distinct predictive profiles. Integrating clinical, dosimetric, immunological, and genetic factors may improve personalized radiotherapy, although prospective multicenter validation remains necessary.

## 1. Introduction

PC represents one of the most frequently diagnosed malignancies in the male population worldwide and continues to pose a significant public health concern because of its considerable impact on patient morbidity and health-related quality of life. Based on the GLOBOCAN 2022 estimates, PC ranked as the second most commonly diagnosed malignancy among men worldwide and the fifth leading cause of cancer-related death, with approximately 1.47 million newly diagnosed cases and almost 397,000 deaths reported in 2022 [1,2]. Its incidence varies considerably across geographic regions, with the highest rates reported in Northern Europe, Australia/New Zealand, the Caribbean, and North America, and the lowest rates observed in Asia and North Africa [1]. Analyses of the Global Burden of Disease dataset covering the period from 1990 to 2021 describe the same geographic gradient, relate it to sociodemographic development, and project a continued rise in the absolute number of cases through 2040 [3,4].

The growing life expectancy of the population, demographic aging, and the broad adoption of early detection approaches, particularly prostate-specific antigen (PSA) screening, have contributed to the continuous increase in PC incidence during recent decades [5,6]. In addition to improving oncological outcomes, reducing treatment-related morbidity, and maintaining patients’ quality of life have become key priorities in the current management of PC. Age–period–cohort analyses restricted to men aged 55 years and older indicate that population aging accounts for a substantial part of this increase [7], and systematic reviews of PC epidemiology summarize the corresponding modifiable and non-modifiable risk factors [8].

External beam radiotherapy (EBRT) represents one of the main therapeutic approaches for PC and is widely employed with curative intent in localized and locally advanced disease, as well as in the adjuvant and salvage settings [9]. Owing to the advances achieved with contemporary radiotherapy techniques, limiting treatment-related toxicity has become an equally important therapeutic objective because of its direct influence on long-term functional outcomes and overall patient quality of life [9]. The clinical context in which these decisions are made is frequently complex. Patients with PC may present with, or subsequently develop, additional primary malignancies, and single-center series of synchronous and metachronous presentations show that such cases require coordinated multidisciplinary planning to reconcile competing treatment demands and cumulative organ doses [10]. Prolonged disease control is achievable in this setting, as illustrated by a reported case of extended progression-free survival managed by a multidisciplinary team [11], which further increases the importance of limiting long-term treatment-related morbidity.

Advances in contemporary radiotherapy techniques have markedly enhanced the accuracy of treatment planning and radiation dose delivery for patients with PC. The implementation of techniques such as IMRT, VMAT, and SBRT has made it possible to achieve improved dose conformity within the target volume while minimizing radiation exposure to adjacent organs at risk, particularly the urinary bladder and rectum [9,12]. These technological developments have enabled the implementation of dose-escalation approaches and hypofractionated treatment schedules while preserving low rates of severe toxicity and an overall favorable safety profile [12,13]. Despite these advances, genitourinary and gastrointestinal toxicities remain clinically relevant even with contemporary radiotherapy techniques, indicating that biological processes and patient-specific susceptibility factors contribute to treatment-related adverse events in addition to the effects of dosimetric parameters alone.

In PC, radiotherapy-related toxicity is traditionally categorized as acute or late based on the timing of its clinical manifestation. Acute toxicity typically occurs during treatment or within the first three months after completion of radiotherapy and is primarily characterized by irritative and obstructive lower urinary tract symptoms, including increased urinary frequency, dysuria, and urinary urgency, together with gastrointestinal manifestations, including diarrhea and rectal tenesmus. Conversely, late toxicity may manifest clinically several months or even years after radiotherapy and comprises persistent or progressive complications, including hematuria, urethral strictures, rectal bleeding, fecal incontinence, and chronic bowel dysfunction [9,14,15]. These complications may significantly compromise long-term functional outcomes, psychological well-being, and patients’ overall quality of life. The psychological burden is not incidental to the functional one: in a hospital cohort of oncology inpatients assessed by psychiatry, urogenital malignancies were associated with psychosocial stressors relating specifically to continence, sexual function, and body image, domains that overlap directly with the endpoints considered in this review [16].

Considering the clinical impact of radiotherapy-related toxicity, identifying predictors of GU and GI adverse events has become a major focus of research in radiation oncology. Current evidence indicates that toxicity risk results from a complex interplay among patient-related characteristics, treatment-related technical and dosimetric parameters, tissue-specific biological responses to ionizing radiation, and individual genetic susceptibility [9,17,18,19]. Nevertheless, the marked interpatient variability in clinical outcomes cannot be fully explained by the clinical and dosimetric variables currently incorporated into routine practice. Accordingly, growing emphasis has been placed on the identification of inflammatory, immunological, and radiogenomic biomarkers capable of improving individualized risk stratification and supporting the development of integrated predictive models. This evolving approach underpins the concept of personalized radiotherapy, in which treatment is customized on the basis of each patient’s biological profile and personalized risk of treatment-related toxicity [9,14,17].

The present narrative review synthesizes the current evidence regarding the clinical, dosimetric, immunological, and genetic determinants of acute and late genitourinary and gastrointestinal toxicities after contemporary radiotherapy for PC. The principal categories of predictors linked to GU and GI toxicities are examined separately, with particular emphasis on the available evidence supporting individualized risk stratification and the development of predictive models applicable to clinical practice. Although numerous risk factors have been identified, the existing evidence remains fragmented, and integrated models simultaneously incorporating clinical, dosimetric, and biological variables are still insufficiently developed. These limitations underscore the need for additional prospective studies and robust external validation strategies to facilitate the implementation of risk-adapted, personalized radiotherapy.

## 2. Materials and Methods

### 2.1. Review Design

This study was conducted as a narrative literature review with the main objective of identifying, critically appraising, and integrating the available evidence on predictive factors for acute and late GU and GI toxicities in patients undergoing EBRT for PC.

**Figure 1:**
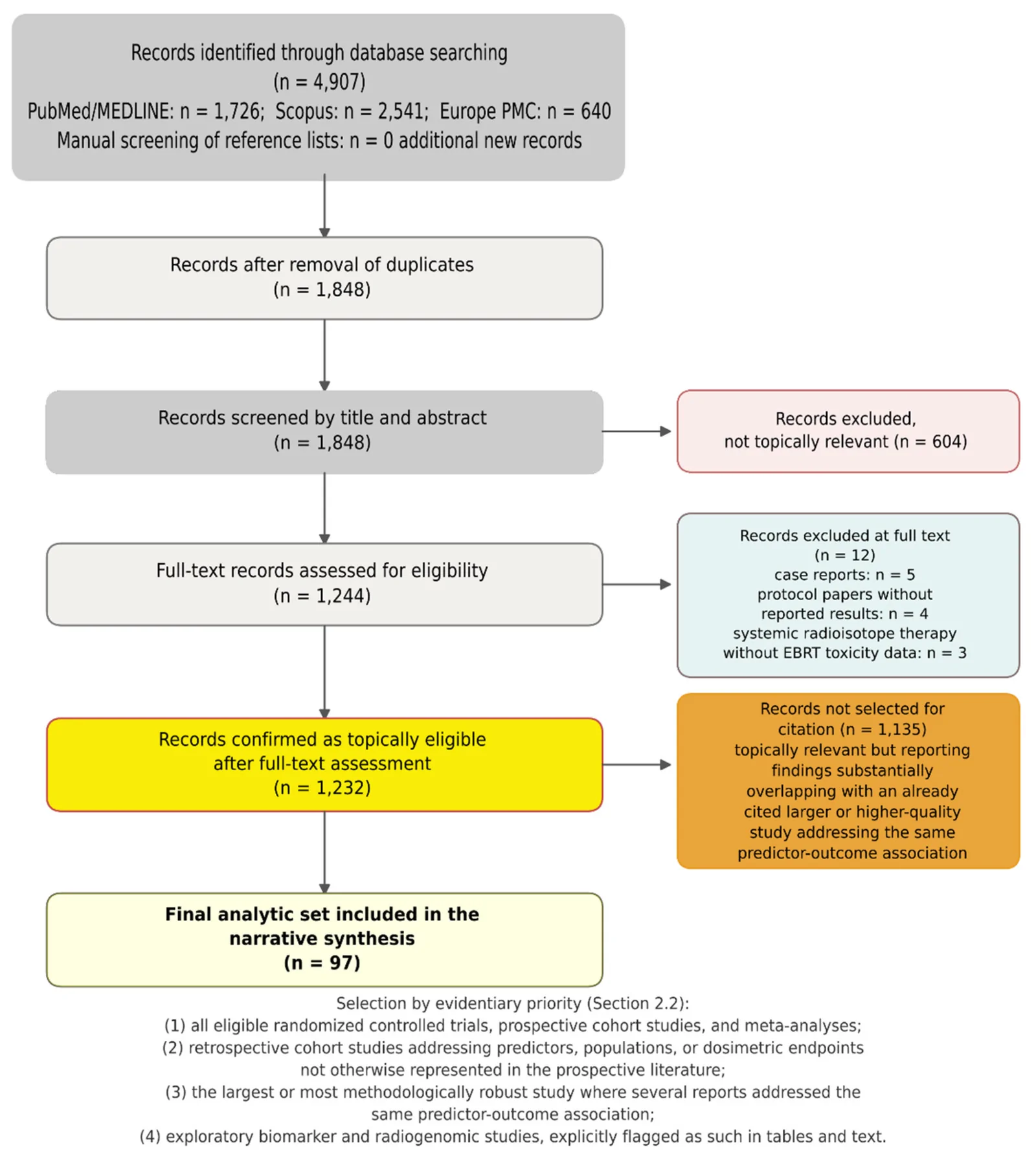
Records passing title and abstract screening underwent full-text assessment, at which stage 12 records were excluded (case reports, *n* = 5; protocol papers without reported results, *n* = 4; systemic radioisotope therapy without external beam radiotherapy toxicity data, *n* = 3). Of the 1232 records confirmed as topically eligible at full text, 97 constitute the final analytic set included in the narrative synthesis, selected according to the evidentiary priority criteria described in Section 2.2. The remaining 1135 records were topically relevant and methodologically acceptable but were not cited, in almost all cases because they reported findings substantially overlapping with an already-cited larger or higher-quality study addressing the same predictor–outcome association.

A narrative review design was selected for substantive methodological reasons. The body of evidence on predictive factors for radiation-induced toxicity in prostate cancer is characterized by marked heterogeneity in study populations, radiotherapy techniques and fractionation schedules, dosimetric parameters evaluated, biomarker assessment methods, toxicity grading systems (RTOG, CTCAE versions 3.0–5.0, and patient-reported outcomes), and duration of follow-up. This heterogeneity precludes quantitative pooling and renders a formal systematic review with a meta-analysis methodologically inappropriate for the scope of this work. Consequently, prospective registration on PROSPERO, which is intended for reviews with a predefined quantitative synthesis plan, was not pursued; methodological transparency was instead prioritized through a fully documented search strategy, structured eligibility criteria, and a complete study selection flow diagram (Section 2.2, Figure 1, Supplementary Table S1). A narrative synthesis was therefore chosen as the framework best suited to critically appraising and integrating evidence spanning clinical, dosimetric, immunological, and radiogenomic domains, while preserving the contextual interpretation necessary for evaluating preliminary and exploratory findings alongside more established associations.

### 2.2. Literature Search Strategy

A structured search of the literature was performed using the PubMed/MEDLINE, Scopus, and Europe PMC databases. PubMed/MEDLINE, Scopus, and Europe PMC together provide extensive and substantially overlapping coverage of the biomedical and oncology literature relevant to this review. The literature search was carried out between January and March 2026, and the search strategy was subsequently reconstructed and formally documented in August 2026 using identical terms and eligibility criteria; records published between March and August 2026 were additionally captured and screened. The overall search covered studies published from 2000 through 2026.

The search strategy combined Medical Subject Headings (MeSH, for PubMed/MEDLINE) with title/abstract free-text keywords adapted to the controlled-vocabulary syntax of each database. The principal search terms included: “prostatic neoplasms,” “prostate cancer,” “radiotherapy,” “radiation therapy,” “intensity-modulated radiotherapy,” “IMRT,” “volumetric modulated arc therapy,” “VMAT,” “stereotactic body radiotherapy,” “SBRT,” “genitourinary toxicity,” “urinary toxicity,” “gastrointestinal toxicity,” “rectal toxicity,” “predictive factors,” “risk factors,” “dose-volume histogram,” “dosimetric parameters,” “cytokines,” “inflammatory biomarkers,” “lymphocyte,” “lymphopenia,” “radiogenomics,” “genetic polymorphism,” “single nucleotide polymorphism,” and “microRNA.” Boolean operators AND and OR were applied to combine terms, with NOT used to exclude pediatric populations and non-prostate malignancies. The complete search strings for each database are provided in Supplementary Table S1. Records were screened in two stages. In the first stage, titles and abstracts were evaluated against the predefined eligibility criteria. In the second stage, full texts of potentially eligible records were retrieved and assessed. Electronic database searches were supplemented by manual screening of reference lists from included publications and relevant review articles. This manual screening identified two additional studies published before the 2000 cutoff applied to the electronic search (Forman et al., 1999; Louagie et al., 1999), which were retained given their direct relevance to prostate volume as a predictor of urinary toxicity and to radiation-induced lymphopenia [20,21]. No other studies outside the 2000–2026 window were identified or included [20,21].

Prospective cohort studies randomized controlled trials (RCTs), meta-analyses, and systematic reviews constituted the primary evidence base. Retrospective cohort studies were included when they addressed a predictor or patient population not adequately covered by prospective data. Exploratory studies of immunological and radiogenomic biomarkers were included as supplementary evidence, given the early stage of development of this research area, but are explicitly identified as such in the tables and discussed with appropriate qualification in the text (see Section 2.3). This graduated approach to evidence integration is intended to preserve transparency regarding the strength of individual associations.

Of the 1232 records assessed as topically eligible following title and abstract screening, 97 studies were selected for inclusion in the narrative synthesis. Given the volume of eligible literature and the narrative (non-systematic) design of this review, selection prioritized: (1) all identified RCTs, prospective cohort studies, and meta-analyses meeting eligibility criteria; (2) retrospective cohort studies providing evidence on predictors, patient populations, or dosimetric endpoints not otherwise represented in the prospective literature; (3) the largest or most methodologically robust studies where multiple reports addressed a similar predictor–outcome association, to avoid redundant citation of overlapping findings; and (4) exploratory biomarker and radiogenomic studies illustrating emerging but not yet clinically validated directions, explicitly flagged as such in tables and text. Records not selected for citation were topically relevant but did not meet these prioritization criteria, most commonly because they reported findings substantially overlapping with an already-cited, larger, or higher-quality study addressing the same association.

The 97 studies cited in this review constitute the final analytic set, established after full-text assessment of all records passing title and abstract screening. No further studies were added or removed at any subsequent stage of manuscript preparation. Full-text exclusion was applied for three predefined reasons: publication type not carrying primary outcome data (case reports, *n* = 5), absence of reported results (protocol papers, *n* = 4), and treatment modality outside the scope of the review (systemic radioisotope therapy without external beam radiotherapy toxicity data, *n* = 3). Non-selection of the remaining 1135 topically eligible records reflects the evidentiary priority framework described above rather than a judgement of methodological inadequacy, and this distinction is made explicit in Figure 1.

### 2.3. Toxicity Assessment

Across the included studies, treatment-related toxicity was predominantly assessed using standardized grading systems, primarily the Radiation Therapy Oncology Group (RTOG) criteria and the Common Terminology Criteria for Adverse Events (CTCAE) (versions 3.0–5.0). In most studies, acute toxicity referred to adverse events occurring during radiotherapy or within the first 90 days following treatment completion, whereas late toxicity referred to the onset or persistence of adverse events occurring more than 90 days after completion of radiotherapy.

Differences in toxicity grading systems, evaluation time points, and operational definitions of acute and late toxicity represent a major contributor to heterogeneity across the published literature and limit direct comparison of reported effect sizes. To make the extent of this heterogeneity explicit, we verified the grading instrument, follow-up duration, and fractionation schedule of the studies cited in Table 1, Table 2, Table 3, Table 4 and Table 5. At least twelve distinct clinician-reported grading instruments are in use across these studies, and likely more, since the instrument was not explicitly named in the accessible sources for approximately one-quarter of the cited studies. Several studies apply different instruments to acute and late endpoints within the same cohort, and at least one study uses a patient-reported instrument (IPSS) as its sole endpoint rather than a clinician-graded scale. The operational definition of late toxicity ranges from any event beyond 90 days to a fixed 2-, 3-, or 5-year cumulative incidence, and median follow-up ranges from acute-only reporting to more than 10 years.

Two consequences follow. First, effect estimates reported for the same predictor in different studies frequently do not measure the same underlying quantity, and the magnitudes cited in Table 1, Table 2, Table 3, Table 4 and Table 5 should be read as indicative of direction and approximate strength rather than as pooled or interchangeable values. Second, where the same cohort is assessed under both RTOG and CTCAE criteria, the reported between-arm difference diverges depending on the instrument used [22,23,24], demonstrating that instrument choice is not a neutral methodological detail.

Because this review was not designed as a systematic review, the PRISMA 2020 checklist was not applied in full; however, the selection process followed the transparency principles underlying PRISMA: a structured search across three databases with documented search terms, predefined eligibility criteria, two-stage screening, and a selection flow diagram (Figure 1). A formal risk-of-bias tool (e.g., ROBINS-I, RoB 2) was not applied at the study level; instead, methodological quality is addressed narratively for individual studies and through the differentiation of evidence levels in tables and text. This approach is consistent with published guidance on narrative reviews that incorporate a structured search component without meeting criteria for a systematic review (e.g., the SWiM reporting guideline for narrative synthesis).

### 2.4. Eligibility Criteria

Study eligibility was determined using predefined inclusion and exclusion criteria.

**Inclusion criteria:**

- Prospective and retrospective clinical investigations randomized controlled trials, meta-analyses, and systematic reviews involving patients who underwent EBRT for PC.
- Studies evaluating predictors of GU and/or GI toxicities, including clinical, dosimetric, immunological, and genetic determinants.
- Studies evaluating treatment-related toxicity according to standardized grading systems, including the RTOG criteria, the CTCAE, or equivalent validated classification systems.
- Publications available in the English language.
- Publications published from 2000 through 2026, identified through the database search. Two earlier studies identified through manual screening of reference lists were retained for their direct relevance to specific predictive factors: Forman et al. (1999), addressing prostate volume as a predictor of urinary morbidity, and Louagie et al. (1999), addressing radiation-induced lymphopenia [20,21]. Both and are explicitly flagged as historical evidence in the corresponding sections.

**Exclusion criteria:**

- Studies lacking specific data on radiotherapy-associated GU or GI toxicity;
- Studies exclusively involving malignancies other than PC;
- Studies evaluating brachytherapy as the sole treatment modality without direct relevance to external beam radiotherapy;
- Studies evaluating systemic radioisotope therapies (e.g., strontium-89, radium-223, lutetium-177) as the primary treatment modality, without EBRT-related toxicity data;
- Studies focused exclusively on erectile or sexual dysfunction outcomes, which fall outside the GU toxicity definition applied in this review;
- Editorials, commentaries, correspondence, and individual case reports;
- Articles published in languages other than English.

The study selection process consisted of two sequential stages. Initially, titles and abstracts were evaluated to identify potentially eligible publications (*n* = 1244 retained); subsequently, these articles underwent full-text evaluation to verify their relevance to the review objectives, at which stage 12 records were excluded on predefined grounds (Figure 1). The 1232 records confirmed as topically eligible at full text formed the pool from which the final analytic set of 97 cited studies was drawn.

## 3. Included Studies and Comparative Analysis of GU and GI Toxicity

The evidence included in this review revealed substantial variability with respect to the reported incidence of GU and GI toxicities. This heterogeneity reflects differences in radiotherapy techniques, fractionation schedules, patient populations, toxicity grading systems, and duration of follow-up across the available literature. For this reason, the incidences presented in Table 1 are reported for each study under its own grading instrument and follow-up interval and are not comparable as absolute rates between studies. Throughout this section, quantitative comparisons are drawn only between arms of the same trial, where grading instrument, ascertainment schedule, and follow-up are shared; comparisons between studies are restricted to the direction and consistency of the reported associations.

### 3.1. Overall Incidence of GU and GI Toxicity

The largest available estimate comes from an individual patient data meta-analysis of six randomized trials (Table 1) [17]. Two features of that dataset are worth noting beyond the incidence figures themselves: within this pooled cohort, graded under a single instrument, acute GU toxicity occurs at roughly twice the rate of acute GI toxicity, whereas the two converge in the late setting, and more than 95% of late grade ≥ 2 events were first documented beyond six months after treatment, which sets a minimum follow-up threshold below which late toxicity is systematically underascertained.

Evidence from individual randomized trials further supports these observations, indicating that toxicity incidence is affected by both the radiotherapy technique and the prescribed radiation dose. Within the randomized RTOG 0126 trial, which compared conventional-dose radiotherapy (70.2 Gy) with high-dose radiotherapy (79.2 Gy) among 1499 patients with intermediate-risk PC, dose escalation increased late toxicity in both domains, with a proportionally larger relative increase for GU than for GI toxicity [25]. Subsequent analyses of the same trial demonstrated that IMRT significantly lowered the occurrence of acute GI and GU toxicities relative to three-dimensional conformal radiotherapy (3D-CRT). Furthermore, multivariable analysis identified a rectal V70 ≥ 15% as an independent factor associated with late grade ≥ 2 rectal toxicity [26].

### 3.2. Impact of Radiotherapy Technique and Fractionation

Randomized trials evaluating moderate hypofractionation have consistently demonstrated a late toxicity profile comparable to that of conventionally fractionated radiotherapy. The CHHiP trial found no significant difference in late grade ≥ 2 toxicity between conventional and moderately hypofractionated arms [13]. These findings were subsequently reinforced by the joint ASTRO/ASCO/AUA clinical practice guideline, which concluded that moderate hypofractionation is linked to a risk of late GU and GI toxicities similar to that observed with conventional fractionation, despite a slight increase in acute GI toxicity rates [12]. In contrast, the HYPRO trial, which compared 39 fractions of 2 Gy with 19 fractions of 3.4 Gy, could not demonstrate non-inferiority of hypofractionation for the cumulative incidence of late grade ≥ 2 GU toxicity (HR 1.16; 90% CI 0.98–1.38) or late grade ≥ 2 GI toxicity (HR 1.19; 90% CI 0.93–1.52), and reported a significantly higher cumulative incidence of late grade ≥ 3 GU toxicity in the hypofractionated arm (19.0% versus 12.9%; *p* = 0.021) [27]. Moderately hypofractionated schedules are therefore not interchangeable, and toxicity outcomes depend on the specific dose per fraction and total dose employed. Ten-year outcomes have also been reported for a more markedly hypofractionated schedule of 45 Gy in 9 fractions, although in a single-institution series without a randomized comparator [28].

With regard to ultrahypofractionation, the HYPO-RT-PC trial found no significant difference between ultrahypofractionation and conventional fractionation at 10 years [29]. This is the longest follow-up available for any ultra-hypofractionated regimen and the only dataset in which late toxicity has been observed beyond the interval at which most trials close.

Results from studies evaluating SBRT indicate an overall favorable toxicity profile, although with distinct characteristics regarding urinary toxicity. The PACE-B trial, representing the first phase III randomized study comparing five-fraction SBRT with conventionally fractionated or moderately hypofractionated radiotherapy, found a significantly higher cumulative incidence of late grade ≥ 2 GU toxicity with SBRT, but no significant difference in 5-year point prevalence (Table 1) [23]. The divergence between these two measures indicates that the excess urinary toxicity associated with SBRT was predominantly transient and largely attributable to a temporary exacerbation of urinary symptoms occurring approximately 12 months after treatment [23].

The findings of the PACE-C trial were consistent with these observations, showing slightly higher acute toxicity after SBRT than after moderate hypofractionation in both domains (Table 1) [22]. Collectively, the available evidence indicates that modern hypofractionated and ultrahypofractionated radiotherapy regimens do not result in a clinically meaningful increase in severe late toxicity. Instead, these treatment approaches appear to modify the temporal pattern and clinical presentation of treatment-related adverse events rather than their long-term overall incidence.

### 3.3. Association Between Acute and Late Toxicity

Evidence from multiple studies consistently indicates that the development of acute toxicity is one of the strongest predictive factors of subsequent late toxicity after radiotherapy for PC. In the IPD meta-analysis, acute grade ≥ 2 toxicity approximately doubled the odds of late toxicity in the corresponding domain (GU: OR 2.20, 95% CI 1.88–2.57; GI: OR 2.53, 95% CI 2.07–3.08; both *p* < 0.0001) [17].

These observations are in agreement with the post hoc evaluation of the PACE-B trial, which showed that acute grade ≥ 2 GU toxicity showed a significant association with late grade ≥ 2 GU toxicity after both SBRT (OR 4.63; 95% CI 2.96–7.25; *p* < 0.0001) and conventionally fractionated radiotherapy (OR 2.83; 95% CI 1.69–4.71; *p* < 0.0001). The same pattern held for GI toxicity in both arms (Table 1) [30]. In addition, the presence of baseline GU symptoms was identified as an independent factor associated with a significantly increased risk of late GU toxicity [30].

Analyses derived from the RTOG 0126 trial further confirmed the contribution of both clinical and therapy-related factors to subsequent late toxicity. Pre-existing urinary dysfunction and radiation dose escalation were identified as independent predictors of an increased likelihood of late GU toxicity, whereas acute GI toxicity, together with higher radiation doses were linked to an increased risk of late GI toxicity [25,26]. In contrast, the use of IMRT was mainly linked to reduced GI toxicity, while its effect on late GU toxicity did not reach statistical significance [26]. A subsequent conditional risk analysis indicated that the likelihood of developing grade ≥ 2 toxicity progressively decreased over time among patients who remained free of treatment-related adverse events following radiotherapy. Specifically, at five years after treatment, the conditional likelihood of developing grade ≥ 2 toxicity over the subsequent five-year period was only 3.02% for GU toxicity and 1.54% for GI toxicity [31].

The Dutch dose-escalation trial identified previous abdominal surgery and baseline GI symptoms as independent predictors of late GI toxicity, and ADT, baseline GU symptoms, and prior TURP as predictors of late GU toxicity (Table 1) [32]. These predictors are examined individually in Section 4.1.

These observations support the existence of a continuum between early and late radiation-related adverse events, described in the literature as “consequential late toxicity”. This concept suggests that strategies aimed at reducing acute toxicity—such as aggressive margin reduction using MRI guidance, urethral-sparing approaches, and perirectal spacers—may also contribute to lowering the risk of subsequent late toxicity [17].

### 3.4. Synthesis of Data from the Main Trials

The principal characteristics of the studies analyzed are summarized in Table 1.

**Table 1:** Overview of the main clinical trials and studies evaluating GU and GI toxicity following radiotherapy for PC.

Study	Study Design	Patients (*n*)	Radiotherapy Technique	Median Follow-Up	Grade ≥ 2 GU Toxicity	Grade ≥ 2 GI Toxicity	Grading Instrument; Follow-Up	Main Findings
Nikitas et al., 2025 [17]	Individual patient data (IPD) meta-analysis	6593	CF + MHF	72 months	29.8% acute; 15.4% late	14.7% acute; 14.3% late	RTOG; median FU 72 mo (IQR 61–94)	Acute toxicity was strongly associated with the subsequent development of late toxicity.
RTOG 0126 (Michalski et al., 2018) [25]	Randomized controlled trial	1499	3D-CRT/IMRT	8.4 years	7–12% late	15–21% late	NCI CTC v2.0 (acute) + RTOG/EORTC (late); median FU 8.4 y	Dose escalation increased late toxicity; IMRT demonstrated a protective effect.
RTOG 0126—Toxicity analysis (Michalski et al., 2013) [26]	Secondary analysis	748	3D-CRT vs. IMRT	3.6 years	No significant difference between techniques	Reduced with IMRT	CTC v2.0 (acute) + RTOG/EORTC (late); median FU 8.4 y (RTOG 0126 cohort)	Rectal V70 ≥ 15% independently predicted late GI toxicity.
CHHiP (Dearnaley et al., 2016) [13]	Randomized controlled trial	3216	IMRT	62.4 months	9.1–11.7% late	11.9–13.7% late	RTOG + EPIC; median FU 62.4 mo	Moderate hypofractionation demonstrated non-inferiority compared with conventional fractionation.
HYPO-RT-PC 10-year follow-up (Nilsson et al., 2026) [29]	Randomized controlled trial	1180	3D-CRT/IMRT	10.6 years	28–30% late	14% late	RTOG; median FU 10.6 y (CF)/10.7 y (UHF)	Ultrahypofractionation remained non-inferior after 10 years.
PACE-B 5—year analysis (van As et al., 2024) [23]	Randomized controlled trial	874	SBRT vs. conventional radiotherapy	5 years	7.3% (SBRT) vs. 4.5% (CRT) (5-year point prevalence)	0.8% vs. 0.3%	RTOG and CTCAE; median FU 74.0 mo	Higher cumulative incidence of late GU toxicity following SBRT.
PACE-C (Tree et al., 2025) [22]	Randomized controlled trial	1185	SBRT vs. moderate HF	Acute toxicity assessment	34% vs. 28% acute	17% vs. 10% acute	RTOG and CTCAE; early toxicity at 12 wk	SBRT was linked to a slight increase in acute toxicity.
PACE-B—Acute-to-late toxicity association (Ratnakumaran et al., 2023) [30]	Post hoc analysis	842	SBRT/conventional radiotherapy	2 years	OR 4.63 (SBRT)	OR 3.67 (SBRT)	CTCAE v4.0; acute toxicity to 12 wk, late toxicity to 2 y (PACE-B cohort)	Acute toxicity strongly predicted subsequent late toxicity.
RTOG 0126—Conditional risk analysis (Alexander et al., 2024) [31]	Secondary analysis	1499	3D-CRT/IMRT	8.4 years	Declining conditional risk	Declining conditional risk	NCI CTC v2.0 (acute) + RTOG/EORTC (late); median FU 8.4 y	Baseline IPSS and radiation dose predicted toxicity; IMRT reduced GI toxicity.
Peeters et al., 2005 [32]	Randomized controlled trial	669	3D-CRT	31 months	28.5–30.2% late	23.2–26.5% late	RTOG (acute) + RTOG/EORTC modified (late); median FU 31 mo	Previous TURP, abdominal surgery, and baseline symptoms were independent predictors of late toxicity.

**Note:** CF = conventional fractionation; MHF = moderate hypofractionation; HF = hypofractionation; NS = not statistically significant; CRT = conventionally fractionated radiotherapy; MHRT = moderately hypofractionated radiotherapy. Incidence figures in this table are reported under different grading instruments, different operational definitions of late toxicity, and follow-up durations ranging from 12 weeks to more than 10 years, as detailed in the “Grading instrument; follow-up” column. They are presented to characterise each individual study and must not be compared as absolute rates across rows. Where the same cohort has been graded under both RTOG and CTCAE criteria, the reported incidence has differed materially depending on the instrument used.

The available evidence demonstrates substantial variability in the occurrence of GU and GI toxicities, reflecting the complex interplay among radiotherapy technique, fractionation schedule, delivered radiation dose, and individual patient characteristics. Overall, modern radiotherapy techniques, particularly IMRT together with SBRT, have enabled lower radiation exposure to organs at risk while maintaining low rates of GI toxicity, thereby improving the overall safety profile of PC radiotherapy. Nevertheless, GU toxicity remains a clinically relevant challenge, particularly in the setting of ultrahypofractionation and SBRT, where a transient exacerbation of urinary symptoms occurring approximately 12–18 months after treatment has been consistently reported [23,29].

The biological mechanisms responsible for the progression from acute to late toxicity have not yet been fully elucidated and continue to represent an active area of investigation. The marked interindividual variability in treatment response suggests that additional determinants—including clinical, immunological, and genetic factors—contribute to toxicity susceptibility and warrant further consideration. These factors are examined in detail in the sections that follow.

## 4. Predictive Factors for GU and GI Toxicity

Predictors of radiotherapy-associated toxicity in PC may be grouped into four main categories: clinical factors, dosimetric factors, immunological and inflammatory biomarkers, and genetic/radiogenomic factors. This section critically reviews each category by distinguishing predictors specific to GU toxicity from those related to GI toxicity, according to the currently available evidence.

### 4.1. Clinical Predictors

Baseline clinical characteristics are major determinants of individual susceptibility to radiotherapy-induced toxicity in patients with PC. Together with technical and dosimetric treatment parameters, these factors contribute substantially to the overall likelihood of developing GU and GI complications. Among the most extensively investigated clinical predictors are the severity of baseline urinary symptoms, patient age, prostatic volume, previous TURP, smoking status, metabolic comorbidities, and the use of urinary medications before treatment [33].

#### 4.1.1. Clinical Predictors of GU Toxicity

(a) International Prostate Symptom Score (IPSS)

The pretreatment IPSS is the most consistently validated and robust clinical predictor of GU toxicity, with its prognostic value confirmed across multiple prospective and retrospective cohorts. A Swedish population-based study including 4436 patients treated with radiotherapy between 2018 and 2023 identified a clear dose–response association between baseline IPSS and urinary toxicity. The 3-year incidence of urinary events was 19% among patients with mild IPSS (0–7), 28% among those with moderate IPSS (8–19), and 39% among those with severe IPSS (20–35). Furthermore, each 5-point increase in the baseline IPSS corresponded to a 20% increase in the risk of urinary events, and this association remained consistent across all predefined subgroups, including prostate volume, radiotherapy modality, and androgen deprivation therapy (ADT) use [34].

The predictive factor analysis from the PACE-B trial, published in European Urology, further confirmed baseline IPSS as an independent predictor of late grade ≥ 2 GU toxicity after SBRT (OR 1.11 per point; 95% CI 1.05–1.18; *p* = 0.001) [35]. Similarly, Malik et al. showed that patients with a baseline IPSS ≥ 15 experienced a significantly higher rate of acute grade ≥ 2 GU toxicity (64% vs. 42%; *p* = 0.0005) and a lower 4-year freedom from late grade ≥ 2 GU toxicity (38% vs. 64%; *p* < 0.0001) [36].

(b) Age

Age ≥ 75 years has been identified as an independent predictive factor for acute grade ≥ 2 GU toxicity in a cohort of 326 patients treated with proton beam therapy (OR 1.80; 95% CI 1.10–2.95; *p* = 0.03). In another cohort comprising 605 patients treated with proton therapy, age, analyzed as a continuous variable, independently predicted acute grade ≥ 2 GU toxicity (HR 1.04; *p* = 0.046), whereas baseline IPSS was the only independent predictive factor for late grade ≥ 2 GU toxicity (HR 1.06; *p* = 0.049) [37,38]. Several other studies did not identify a significant association between chronological age and moderate or severe GU toxicity [33,37,38]. The inconsistency is unsurprising, since age is correlated with several of the other predictors: older men have higher baseline IPSS, larger prostate volumes, a greater likelihood of prior TURP, and a higher burden of cardiovascular comorbidity and anticoagulant use. Studies that adjust for baseline urinary function generally attenuate or lose the age effect, which suggests that chronological age acts largely as a proxy for these factors rather than as an independent determinant of radio sensitivity. Age should therefore not be used as a standalone risk criterion, and should not by itself preclude the use of hypofractionated regimes in otherwise suitable patients.

(c) Prostate Volume

An enlarged prostate volume has been linked to a higher likelihood of GU toxicity in numerous studies, particularly during the acute phase following radiotherapy, although the available evidence is not entirely consistent across different treatment modalities and fractionation schedules. A study involving 214 patients undergoing IMRT demonstrated that for each 27 cm^3^ increase in prostate volume, the probability of acute grade 3 GU toxicity doubled (*p* = 0.006 in multivariable analysis) [39]. In the setting of SBRT, a prostate volume threshold of 60 cm^3^ was recognized as a significant predictive factor for acute grade 3–4 GU toxicity [40]. Pinkawa et al. further found that patients with larger prostate volumes had a greater probability of experiencing acute irritative and obstructive urinary symptoms, with daily dysuria occurring in 48% versus 18% of patients (*p* < 0.01). However, these symptoms were resolved rapidly and did not negatively influence long-term quality of life [41]. Comparable clinical determinants of urinary morbidity have also been examined in men receiving combination external beam radiotherapy with a brachytherapy boost [42].

(d) History of transurethral resection of the prostate (TURP)

The history of TURP is recognized as a well-established risk factor for late GU toxicity following radiotherapy. A systematic review demonstrated that prior TURP is associated with a higher risk of late GU toxicity after prostate radiotherapy, although substantial heterogeneity among the included studies precluded a quantitative synthesis [33]. Byrne et al. reported that, in a multivariable analysis of a cohort of 300 patients treated with IMRT to doses of 78–84 Gy, previous TURP was the only factor that remained independently associated with late grade ≥ 2 GU toxicity (HR 2.54; *p* = 0.002) [43]. A more recent study with a median follow-up of 78 months confirmed that prior TURP was independently linked to a higher incidence of late grade ≥ 2 GU toxicity (21.5% vs. 7.6%; *p* = 0.02) as well as an earlier onset of toxicity (16 vs. 47 months; *p* = 0.007) [44]. In addition, a scoping review identified late hematuria as the most commonly reported adverse event among patients with a history of TURP who subsequently underwent hypofractionated radiotherapy [45].

(e) Smoking

Smoking has consistently been linked to a higher risk of late GU toxicity after prostate radiotherapy. In a study including 2156 patients managed at the Memorial Sloan Kettering Cancer Center, current smokers exhibited a significantly higher risk of late GU toxicity (HR 1.80; *p* = 0.02), while former smokers also demonstrated an elevated risk (HR 1.45; *p* = 0.01). In contrast, smoking showed no association with an increased risk of GI toxicity [46]. Stankovic et al. further confirmed active smoking as an independent predictive factor of acute grade ≥ 2 GU toxicity in multivariable analysis (*p* = 0.003) [47]. Likewise, Cozzarini et al. identified smoking as a strong predictor of several acute urinary symptoms, including incomplete bladder emptying, increased urinary frequency, intermittency, urgency, and straining during voiding [48]. The biological rationale for these associations is well characterized: tobacco exposure produces microvascular injury, tissue hypoxia, and impaired repair capacity, and continued smoking during treatment has been linked to poorer outcomes across cancer types treated with radiotherapy [49]. Structured smoking cessation support is accordingly recommended for all patients with cancer undergoing active treatment [50].

(f) Diabetes Mellitus

Diabetes mellitus has been linked to GU toxicity across several studies. Kopčalić et al. reported a significant positive association between diabetes and changes in acute GU toxicity grade severity in radiotherapy [51]. The proposed biological mechanisms include microvascular injury, impaired tissue perfusion, and delayed tissue repair, all of which may increase tissue susceptibility to radiation-induced damage [33]. The magnitude of the reported effect varies considerably across cohorts [33,43]. Interpretation is complicated by the fact that diabetes rarely occurs in isolation: it clusters with hypertension, cardiovascular disease, and anticoagulant or antiplatelet use, each of which is independently associated with toxicity. Few of the available analyses adjust simultaneously for these covariates, so the extent to which diabetes contributes independently, as opposed to marking a broader vascular comorbidity phenotype, remains unresolved. Beyond the vascular domain, type 2 diabetes in patients with cancer has been associated with poorer functional status and with markers of systemic and neuroinflammatory activation [52], which suggests that the comorbidity clusters relevant to toxicity risk may extend further than the microvascular mechanism usually invoked. That work did not assess radiation-induced toxicity, so the connection remains a hypothesis rather than an established mechanism. Glycemic control, disease duration, and the presence of established microvascular complications are plausible effect modifiers but have not been systematically evaluated in this setting.

(g) Pretreatment Urinary Medication (Alpha-Blockers)

Pretreatment use of alpha-blockers has been identified as a predictive factor for GU toxicity in several studies. The PACE-B analysis demonstrated that baseline use of urinary medication was independently linked to late-grade ≥ 2 GU toxicity (OR 2.32; 95% CI 1.09–4.95; *p* = 0.03) [35]. This was confirmed by Byrne et al. in univariable analysis [43]. However, in most studies, alpha-blocker use is considered a surrogate marker of pre-existing lower urinary tract symptoms and baseline voiding dysfunction rather than an independent causal determinant of radiation-induced GU toxicity [35,43].

Overall, the available evidence indicates that the severity of baseline urinary symptoms, as quantified by the IPSS, remains the most consistent clinical predictor of GU toxicity following PC radiotherapy. Additional factors—including a history of TURP, increased prostate volume, smoking, and diabetes mellitus—further contribute to the individual risk of toxicity, although the magnitude of these associations varies among studies. Collectively, these findings underscore the importance of a comprehensive pretreatment clinical assessment, which should subsequently be integrated with dosimetric and biological parameters within multifactorial predictive models to optimize individualized toxicity risk stratification.

#### 4.1.2. Clinical Predictors of GI Toxicity

Compared with GU toxicity, a smaller number of clinical variables have been consistently recognized as predictors of GI toxicity after prostate radiotherapy. However, accumulating evidence has demonstrated that several patient-related factors are consistently linked to an elevated likelihood of late rectal bleeding, fecal incontinence, and other delayed GI complications. These factors include anticoagulant therapy, cardiovascular comorbidities, previous abdominal surgery, and specific anthropometric patient characteristics [33].

(a) Anticoagulant and Antiplatelet Therapy

Anticoagulant therapy has consistently been recognized as an important clinical predictor of late rectal bleeding after prostate radiotherapy. Schreiber et al. reported that patients receiving warfarin or clopidogrel during radiotherapy had a significantly greater risk of rectal bleeding (HR 4.84; 95% CI 1.84–12.68; *p* = 0.001), whereas initiation of anticoagulant treatment after radiotherapy completion did not significantly affect this risk (HR 0.78; *p* = 0.71) [53]. Likewise, Choe et al. found that the 4-year actuarial rate of grade ≥ 3 hemorrhagic toxicity was 15.5% in patients treated with anticoagulants, compared with 3.6% in those who did not receive anticoagulant therapy (*p* < 0.0001) [54]. Kim et al. also identified anticoagulant use as an independent risk factor for late-grade ≥ 3 rectal bleeding (HR 2.93; 95% CI 1.14–7.55; *p* = 0.026) [55]. Furthermore, Musunuru et al. reported anticoagulant therapy to be among the strongest clinical predictors of late hematochezia following SBRT (OR 6.5) [56].

(b) Cardiovascular Comorbidities

Several studies have described a relationship between cardiovascular comorbidities and a higher likelihood of late GI toxicity, particularly radiation-induced rectal bleeding. Bae et al. reported cardiovascular disease as an independent risk factor for rectal bleeding (HR 2.73; *p* = 0.037) [57]. Cicchetti et al. subsequently confirmed, in a cohort of 1633 patients, that both cardiovascular disease and previous abdominal surgery represent independent risk factors for late rectal bleeding [58]. Furthermore, Schaake et al. identified cardiovascular disease, together with bladder wall V75, as an independent predictor of late hematuria, indicating that the influence of cardiovascular comorbidities on radiation-related toxicity may not be limited to GI complications [59].

(c) Body Mass Index (BMI): A Likely Dosimetrically Mediated Association

A seemingly paradoxical observation was described by Schaldemose et al. (2025) in a prospective study including 248 patients, where increased BMI was linked to a lower risk of rectal bleeding [60]. Compared with patients of normal weight, those with a BMI of 25–29.9 kg/m^2^ showed a significantly lower risk (HR 0.54; *p* = 0.044), whereas individuals with a BMI > 29.9 kg/m^2^ experienced an even greater reduction in risk (HR 0.40; *p* = 0.008) [60]. These results were further supported by De Bruyn et al., who reported a protective effect of obesity on both patient-reported and clinician-reported GI toxicity (adjusted OR 0.04; *p* = 0.02) [61]. Furthermore, Doi et al. found that patients with a BMI ≥ 25 kg/m^2^ had significantly lower rectal V50 and V60 dose–volume parameters, providing a potential dosimetric explanation for the reduced incidence of GI toxicity, although these patients simultaneously experienced a higher frequency of late GU toxicity [62].

The association is better described as dosimetrically mediated than as paradoxical. Doi et al. showed directly that patients with a BMI ≥ 25 kg/m^2^ received lower rectal V50 and V60 [62], which indicates that increased pelvic adipose tissue displaces the rectum from the high-dose region rather than conferring any biological protection against radiation injury. On this interpretation, BMI is a marker of favourable pelvic anatomy in a given planning geometry, not an independent determinant of radiosensitivity, and the association would be expected to attenuate or disappear in analyses that adjust for delivered rectal dose. Consistent with a purely geometric explanation, the same study reported a higher frequency of late GU toxicity in the higher-BMI group [62], the opposite of what a general radioprotective mechanism would predict. BMI should therefore not be interpreted as a protective factor in patient counselling, and the finding does not support relaxing rectal constraints in patients with higher BMI.

This interpretation generates a specific, testable hypothesis: increased perirectal and mesorectal adipose tissue volume may act analogously to a rectal spacer, physically displacing the rectal wall away from the high-dose region during treatment. This hypothesis could be tested directly by measuring perirectal fat volume on planning CT or MRI and correlating it with rectal V50/V60 and with the concurrent increase in bladder dose, rather than relying on BMI as an indirect anthropometric surrogate for pelvic fat distribution.

(d) Pretreatment Hemorrhoids and Previous Abdominal Surgery

Pretreatment hemorrhoids have been recognized as independent predictors of rectal bleeding after prostate radiotherapy. Although most available evidence is derived from cohorts treated with external beam radiotherapy, comparable observations have been described in brachytherapy studies, in which pretreatment hemorrhoids were linked to a higher risk of rectal hemorrhage, suggesting an underlying local susceptibility independent of the irradiation technique employed [63]. Musunuru et al. also identified pretreatment hemorrhoids as an independent predictor of late hematochezia following SBRT (OR 2.7) [56]. Previous abdominal surgery was also recognized as an independent risk factor for late grade ≥ 2 GI toxicity in the Dutch dose-escalation trial (*p* < 0.001) [32]. This observation was subsequently externally validated by Cicchetti et al., who likewise identified previous abdominal surgery as an independent predictor of late fecal incontinence [58].

(e) Diabetes Mellitus

Diabetes mellitus has been associated with both GU and GI toxicity in several observational cohorts. The proposed biological mechanisms include radiation-induced susceptibility related to microvascular damage, endothelial dysfunction, impaired tissue repair, and the persistence of a chronic pro-inflammatory state, all of which may potentially cause radiation-induced injury to normal tissues [33,43]. The available evidence remains inconsistent, and the independent contribution of diabetes to GI toxicity is less well established than for GU toxicity. The confounding structure described in Section 4.1.2 applies here with greater force, since anticoagulant use, which is among the strongest predictors of rectal bleeding, is substantially more prevalent among patients with diabetes.

Overall, clinical predictors of GI toxicity are fewer in number and less consistently validated than those associated with GU toxicity. Current evidence consistently supports anticoagulant therapy, cardiovascular comorbidities, and previous abdominal surgery as major risk factors for late rectal bleeding and other delayed GI adverse events. In contrast, the contribution of factors such as body mass index and diabetes mellitus remains insufficiently understood and requires further validation in contemporary prospective cohorts.

#### 4.1.3. Summary Table of Clinical Predictors

The findings summarized in Table 2 demonstrate that the clinical predictors of GU toxicity are both more numerous and more consistently validated than those associated with GI toxicity. Baseline IPSS and a history of TURP represent the most robust clinical predictors of urinary toxicity, whereas anticoagulant therapy, cardiovascular comorbidities, and previous abdominal surgery are among the clinical factors most consistently associated with the development of late GI toxicity (Table 2).

**Table 2:** Clinical predictors of GU and GI toxicity following PC radiotherapy.

Clinical Predictor	Associated Toxicity	Type of Supporting Evidence	Key Findings	References
Elevated pretreatment IPSS	GU (acute and late)	Large prospective cohort; RCT	OR 1.11 per IPSS point (PACE-B); 20% increase per 5-point increase (*n* = 4436)	[34,35,36]
Age ≥ 75 years	GU (primarily acute)	Prospective cohort	OR 1.80 (*p* = 0.03); not confirmed in other cohorts; attenuates after adjustment for baseline urinary function	[37,38]
Prostate volume > 50–60 cm^3^	GU (primarily acute)	Retrospective cohort	Each 27 cm^3^ increase doubled the risk of grade 3 toxicity	[39,40,41]
History of TURP	GU (primarily late)	Systematic review; prospective and retrospective cohorts	HR 2.54 (*p* = 0.002); 21.5% vs. 7.6% incidence of late toxicity	[33,43,44]
Current or former smoking	GU (late)	Large prospective cohort (*n* = 2156)	HR 1.80 for current smokers; HR 1.45 for former smokers	[46,47,48]
Diabetes mellitus	GU and GI	Prospective cohort; heterogeneous evidence	Associated with increased toxicity risk; effect size varies across cohorts; independent contribution uncertain given clustering with cardiovascular comorbidity and anticoagulant use	[33,43,51]
Pretreatment urinary medication (alpha-blockers)	GU (late)	RCT (PACE-B)	OR 2.32 (*p* = 0.03)	[35,43]
Anticoagulant/antiplatelet therapy	GI (rectal bleeding)	Retrospective cohort	HR 4.84 for warfarin/clopidogrel use during radiotherapy	[53,54,55,56]
Cardiovascular disease	GI (rectal bleeding)	Prospective cohort; externally validated NTCP model	HR 2.73 (*p* = 0.037)	[57,58,59]
Body mass index > 29.9 kg/m^2^	GI (inverse association)	Prospective cohort	HR 0.40 (*p* = 0.008); likely mediated by lower rectal V50/V60 rather than biological protection; higher late GU toxicity reported in the same cohort	[60,61,62]
Pretreatment hemorrhoids	GI (rectal bleeding)	Retrospective cohort	OR 2.7 following SBRT	[56,63]
Previous abdominal surgery	GI (late)	RCT; external validation	Independently associated with late GI toxicity (*p* < 0.001)	[32,58]

Type of supporting evidence refers to the study design(s) from which the association was derived, not a formal grading of evidence quality; predictors evaluated within randomized controlled trials reflect observational associations within the trial cohort unless the predictor itself was the randomized variable.

### 4.2. Dosimetric Predictors

Dosimetric parameters provide a quantitative description of how radiation dose is distributed across organs at risk (OARs) during treatment. Their validation status differs substantially between the two toxicity domains. Rectal dose–volume constraints have been derived from randomized trial data, replicated across cohorts, and incorporated into consensus guidelines. Bladder and urethral constraints are considerably less well established: interfraction variation in bladder filling limits the reproducibility of volumetric parameters, published thresholds have not been consistently replicated, and several of the substructure-based associations described below derive from single-institution modelling studies. The sections that follow should be read with this asymmetry in mind. These parameters are commonly expressed using standardized dosimetric metrics, including V_x_ (the percentage of an organ volume receiving at least × Gy), D_x_(the dose received by a specified percentage or volume of an organ), and the mean dose (D_mean_).

#### 4.2.1. Dosimetric Predictors of GI Toxicity

(a) Rectal Dose–Volume Constraints

Data from the RTOG 0126 trial showed that a rectal V70 ≥ 15% was significantly correlated with late grade ≥ 2 rectal toxicity (*p* = 0.034) [26]. A subsequent prospective validation study by Schaldemose et al. demonstrated that maintaining a rectal V74 Gy ≤ 12% is an appropriate dose constraint for reducing patient-reported rectal bleeding, with this target successfully achieved in 99.6% of patients [60].

Among patients treated with five-fraction SBRT, Musunuru et al. identified rectal V38 as the strongest dosimetric predictor of late hematochezia (AUC 0.65). A rectal V38 threshold of ≥2.0 cm^3^ was linked to a substantially higher risk of this complication (OR 4.7) [56]. Likewise, Bae et al. reported rectal wall V65 as an independent predictor of radiation-induced rectal bleeding (HR 1.158; *p* = 0.027) [57].

(b) Radiation Dose to the Iliococcygeus Muscle and the External Anal Sphincter: Predictors of Fecal Incontinence

Recent studies have shown that individual anorectal complications are related to radiation exposure involving specific anatomical substructures. Schaake et al. developed normal tissue complication probability (NTCP) models in a cohort of 262 patients and found that late fecal incontinence was associated with radiation dose delivered to the external anal sphincter (V15) and the iliococcygeus muscle (V55), whereas increased stool frequency correlated with irradiation of the iliococcygeus muscle (V45) and the levator ani muscle (V40) [59]. Smeenk et al. subsequently proposed mean dose constraints aimed at lowering the likelihood of fecal urgency: ≤30 Gy for the internal anal sphincter, ≤10 Gy for the external anal sphincter, ≤50 Gy for the puborectalis muscle, and ≤40 Gy for the levator ani muscle [64].

Alsadius et al., in a cohort of 414 patients, reported that the long-term prevalence of fecal leakage was closely related to the mean radiation dose received by the anal sphincter region, with prevalence increasing significantly at doses ≥ 40 Gy (prevalence ratio 3.8; 95% CI 1.6–8.6) [65]. Furthermore, a recent systematic review estimated that fecal incontinence after PC radiotherapy occurs in 1–12% of patients, depending on the definition adopted, duration of follow-up, and radiotherapy technique [66].

The French ICONES study identified the following independent prognostic factors for late fecal incontinence: a minimum dose of ≥6.4 Gy delivered to the anal canal (OR 2.8; 95% CI 1.03–7.58; *p* = 0.04) and a maximum rectal dose of ≥68.4 Gy (OR 2.4). Conversely, daily image-guided radiotherapy (IGRT) using cone-beam computed tomography (CBCT) at each treatment fraction was reported as an independent protective factor (OR 0.4; *p* = 0.02) [67].

(c) Pelvic Nodal Irradiation

Pelvic nodal irradiation increases radiation exposure to the small bowel and rectum and may consequently increase the risk of GI toxicity. In the randomized POP-RT trial, late grade ≥ 2 GI toxicity occurred more frequently in patients treated with whole-pelvic radiotherapy than in those receiving prostate-only radiotherapy (6.5% vs. 3.8%), although this difference was not statistically significant (*p* = 0.39) [68]. These findings indicate that extending the radiation field to include the pelvic lymph nodes should be considered on an individual basis by weighing the expected oncological benefit against the potential increase in treatment-related GI toxicity [68].

#### 4.2.2. Dosimetric Predictors of GU Toxicity

(a) Radiation Dose to the Bladder Trigone

Ghadjar et al. reported, in a cohort of 268 patients treated with IMRT to a total dose of 86.4 Gy, that the highest radiation dose delivered to the bladder trigone independently predicted clinically significant deterioration in the IPSS, defined as an increase of ≥10 points, on multivariable analysis (*p* = 0.005). Patients with a low baseline IPSS combined with a high maximum trigonal dose had a 29% probability of clinically significant symptom worsening, compared with 7% among those with a high baseline IPSS and a low trigonal dose (OR 5.19; *p* = 0.001) [69]. Schaake et al. also reported that late urinary incontinence was related to the mean radiation dose delivered to the bladder trigone, whereas dysuria correlated with trigonal V75 [59].

Using voxel-based analysis, Mylona et al. identified specific bladder subregions associated with distinct urinary toxicities. Acute urinary retention was associated with the bladder trigone, whereas late urinary retention and dysuria were linked to the posterior bladder wall, and late hematuria to the superior bladder wall. These anatomically defined subregions showed better predictive performance than whole-bladder dosimetric parameters (AUC 0.62–0.81 vs. 0.59–0.72) [70]. A conceptually similar spatially resolved approach has been applied to permanent prostate brachytherapy, in which features of the dose distribution rather than whole-organ dose–volume parameters were used to predict long-term patient-reported urinary toxicity [71]. Brachytherapy as a sole modality falls outside the scope of this review, but the finding supports the more general principle that the spatial distribution of dose carries predictive information not captured by conventional dose–volume histograms.

(b) Radiation Dose to the Urethra

Recent evidence indicates that urethral dosimetry, particularly the $D_{0.1cm^{3}}$ parameter, is linked to the occurrence of grade ≥ 2 GU toxicity, including increased urinary frequency, urinary retention, and urinary incontinence. A systematic review including 23 prospective clinical trials and 2232 patients treated with ultrahypofractionated radiotherapy estimated that keeping the maximum urethral dose below 107% of the prescribed dose (for a regimen of 40 Gy in 5 fractions) lowered the incidence of moderate-to-severe late GU toxicity from 34.3% to 7.7% [72].

(c) Radiation Dose to the Urinary Bladder

Dose–volume constraints for the urinary bladder remain poorly defined. Attempts to establish reliable thresholds have produced inconsistent findings, largely because interfraction variation in bladder filling means that the volume contoured at planning may differ substantially from the volume irradiated at any given fraction, so the dose–volume histogram derived from a single planning scan is an unreliable surrogate for delivered dose [33]. The QUANTEC recommendations of bladder V65 below 50% and V70 below 35% are frequently cited [73], but the underlying evidence base was acknowledged by the QUANTEC authors themselves to be weaker than for the rectum, and these constraints have not been prospectively validated as predictive of GU toxicity in the way that rectal constraints have. The substructure analyses described above, implicating the trigone and specific bladder wall regions, are mechanistically attractive and may partly explain why whole-organ parameters perform poorly, but they derive from single-institution modelling studies without external validation and do not currently constitute planning constraints.

Online adaptive radiotherapy is being investigated as a means of addressing this limitation directly. On magnetic resonance-guided linear accelerators, daily volumetric imaging permits the treatment plan to be re-optimized on the anatomy present at each fraction, and deformable registration of the daily images allows the dose actually delivered to the bladder to be accumulated across the course rather than inferred from a single planning scan. Willigenburg et al. applied this approach in 130 patients treated with SBRT on a 1.5 T MR-Linac and found that accumulated bladder and bladder wall parameters, including V10–35Gy and Dmean, were associated with a clinically relevant increase in IPSS or initiation of alpha-blockers within three months (odds ratios 1.04 to 1.30 after adjustment for baseline characteristics) [74]. A dosimetric comparison of adapted and non-adapted delivery for ultrahypofractionated prostate radiotherapy on the same platform has quantified the extent to which daily re-optimization alters organ-at-risk dose relative to non-adaptive image-guided delivery [75]. These data indicate that the weak performance of bladder dose–volume constraints derived from planning scans may be partly an artefact of dose estimation rather than an absence of a true dose–response relationship. Whether accumulated-dose parameters will yield prospectively validated bladder constraints remains to be established, since the available analyses are single-institution, retrospective in their dosimetric reconstruction, and limited to acute patient-reported endpoints.

#### 4.2.3. Perirectal Spacers

Injectable perirectal hydrogel spacers provide temporary anatomical separation between the prostate and the rectum, resulting in lower rectal radiation exposure and improved achievement of rectal dose–volume objectives. Clinical evidence suggests that their use is associated with reduced GI toxicity following prostate radiotherapy [15].

#### 4.2.4. Summary Table of Dosimetric Predictors

The findings summarized in Table 3 demonstrate that GI toxicity is predominantly determined by rectal dose–volume parameters and radiation exposure to specialized anorectal substructures, including the anal sphincters, anal canal, and pelvic floor musculature. In contrast, GU toxicity is primarily influenced by the radiation dose delivered to the bladder trigone, urethra, and urinary bladder. Collectively, these observations underscore the importance of precise delineation of anatomical substructures and strict adherence to contemporary dosimetric constraints during treatment planning for PC radiotherapy.

**Table 3:** Dosimetric predictors of GU and GI toxicity following PC radiotherapy.

Organ/Structure	Dosimetric Parameter	Validation Level	Associated Toxicity	Key Findings	Grading Instrument; Follow-Up	Ref.
Rectum	V70 ≥ 15%	Secondary analysis of RCT (RTOG 0126)	Late-grade ≥ 2 GI toxicity	*p* = 0.034 (RTOG 0126)	CTC v2.0 (acute) + RTOG/EORTC (late); median FU 8.4 y	[26]
Rectum	V74 ≤ 12%	Prospective cohort (*n* = 248)	Rectal bleeding	Prospectively validated threshold (*n* = 248)	CTCAE + EPIC; median FU 18 mo	[60]
Rectum	V38 ≥ 2.0 cm^3^ (SBRT)	Retrospective cohort	Late hematochezia	OR 4.7 (AUC 0.65)	Institution-specific system; median FU 29.7 mo	[56]
Rectal wall	V65	Prospective cohort; externally validated NTCP model	Rectal bleeding	HR 1.158 (*p* = 0.027)	Vienna Rectoscopy Score; 5 y	[57]
External anal sphincter	V15	Externally validated NTCP model	Fecal incontinence	Validated NTCP model	CTCAE v3.0; follow-up duration not reported	[59]
Iliococcygeus muscle	V55	Externally validated NTCP model	Fecal incontinence	Validated NTCP model	CTCAE v3.0; follow-up duration not reported	[59]
Iliococcygeus muscle	V45	Externally validated NTCP model	Increased stool frequency	Validated NTCP model	CTCAE v3.0; follow-up duration not reported	[59]
Anal sphincter region	Dmean ≥ 40 Gy	Large prospective cohort (*n* = 414)	Fecal leakage	Prevalence ratio 3.8	Study-specific questionnaire (not RTOG/CTCAE)	[65]
Anal canal	Dmin ≥ 6.4 Gy	Prospective cohort (ICONES study)	Fecal incontinence	OR 2.8 (ICONES study)	Jorge & Wexner score; median FU 55 mo	[67]
Bladder trigone	Dmax	Prospective cohort	IPSS deterioration ≥ 10 points	*p* = 0.005; OR 5.19	IPSS (≥10-point increase as endpoint); median FU 5 y	[69]
Urethra	Dmax < 107% of the prescribed dose	Systematic review (23 prospective trials; *n* = 2232)	Moderate-to-severe late GU toxicity	7.7% vs. 34.3%	Grade 2+/3+ noted, instrument not specified; median FU 32 mo	[72]
Urinary bladder	V65, V70	QUANTEC consensus guideline	Late GU toxicity	QUANTEC dose constraints	QUANTEC (dose-constraint framework, not a toxicity scale)	[73]

Effect estimates in this table derive from studies using different grading instruments, endpoint definitions, and follow-up durations, and in several cases from single-institution modelling. They indicate the direction and approximate strength of each dose–volume association and are not interchangeable or poolable across rows. Follow-up duration is stated as reported in the source publication; “not reported” indicates that the information was not available in the accessible source.

### 4.3. Immunological, Inflammatory, and Hematological Biomarkers

The evidence on immunological and inflammatory biomarkers differs in maturity from the clinical and dosimetric evidence reviewed above and should be read accordingly. The studies summarized in this section enrolled between 32 and 214 patients, were conducted at single institutions, and, with the exception of secondary analyses of trial cohorts, were not designed primarily to evaluate toxicity prediction. None of the associations described below has been validated in an independent external cohort, and no biomarker discussed here has an established assay threshold, reference range, or reported discriminatory performance sufficient for clinical decision-making. The findings are presented as directions for prospective investigation rather than as candidates for near-term implementation.

The immune and inflammatory response to ionizing radiation plays a pivotal role in the development of radiation-induced toxicity. Increasing evidence indicates that pro-inflammatory cytokines, systemic inflammatory biomarkers, and alterations in lymphocyte subpopulations may serve as predictors of both GU and GI toxicity. However, most currently available data originate from relatively small and heterogeneous cohorts. Furthermore, the absence of external validation and standardized assessment protocols continues to limit the implementation of these biomarkers in routine clinical practice.

#### 4.3.1. Interleukin-6 (Il-6)

Interleukin-6 (IL-6) is one of the most extensively studied pro-inflammatory cytokines in relation to radiation-induced toxicity after prostate radiotherapy. Stanojković et al. reported, in a cohort of 44 patients treated with three-dimensional conformal radiotherapy (3D-CRT), that elevated serum IL-6 concentrations measured during treatment were significantly associated with greater severity of acute GU toxicity in both univariable and multivariable analyses [76]. Likewise, Kopčalić et al. found, in 39 patients, that both pretreatment IL-6 concentrations and serum IL-6 levels measured after the 25th treatment fraction were positively associated with the highest grade of acute GU toxicity [51]. In contrast, Pinkawa et al. described an apparently paradoxical observation, reporting that serum IL-6 concentrations measured at the T2 time point were significantly associated with the subsequent occurrence of late bowel toxicity (*p* = 0.01), suggesting that both the magnitude and timing of the early inflammatory response may influence the later development of radiation-induced tissue injury [77].

From a biological perspective, IL-6 amplifies the inflammatory response by stimulating the synthesis of acute-phase proteins, promoting the recruitment of inflammatory cells, and activating signaling pathways involved in radiation-induced tissue damage. These processes may contribute to tissue edema, disruption of epithelial integrity, and microvascular dysfunction within the urinary bladder and rectum, thereby increasing susceptibility to radiation-induced toxicity.

#### 4.3.2. Transforming Growth Factor Beta 1 (Tgf-Β1)

TGF-β1 plays a central role in tissue fibrosis and remodeling and is widely recognized as a key mediator of late radiation-induced toxicity. D’Auria et al. reported, in a single-center cohort of 38 patients treated with hypofractionated radiotherapy using a simultaneous integrated boost (SIB), that baseline TGF-β1 concentrations were significantly higher among patients who subsequently developed grade 2–3 late GU toxicity than among those with grade 0–1 toxicity [78]. This observation is hypothesis-generating; the cohort size precludes reliable effect estimation, and the finding has not been replicated in an independent population. Kopčalić et al. likewise reported a positive association between TGF-β1 levels and the severity of acute GU toxicity [51]. Furthermore, Stanojković et al. observed a progressive decline in TGF-β1 gene expression during the course of radiotherapy, suggesting the presence of dynamic regulatory mechanisms in response to radiation exposure [76].

The involvement of TGF-β1 in radiation-induced fibrosis is supported by extensive experimental and clinical evidence. This cytokine promotes fibroblast activation, extracellular matrix deposition, and long-term tissue remodeling, all of which are fundamental processes contributing to the development of late radiation toxicity. The mechanistic rationale for TGF-β1 as a candidate biomarker is therefore well established. Clinical evidence supporting its predictive value in prostate radiotherapy, however, derives from cohorts of fewer than 50 patients without external validation, and does not currently support its use for individual risk stratification.

#### 4.3.3. Tumor Necrosis Factor Alpha (Tnf-A)

TNF-α is a major pro-inflammatory cytokine involved in the cellular response to ionizing radiation. Pinkawa et al. reported that changes in TNF-α levels measured at the completion of radiotherapy (T5) independently predicted late bowel toxicity (*p* = 0.03) [77]. Biologically, TNF-α promotes the recruitment and activation of inflammatory cells, increases vascular permeability, and disrupts intestinal mucosal integrity. These mechanisms are thought to contribute to both acute and late GI toxicity, including diarrhea, rectal tenesmus, and rectal bleeding.

#### 4.3.4. C-Reactive Protein (CRP)

CRP is a nonspecific marker of systemic inflammation, and its role in predicting radiation-induced toxicity is complex and incompletely defined. Pinkawa et al. demonstrated that CRP concentrations > 5 mg/L at the T2 time point (the first assessment during radiotherapy) independently predicted acute urinary toxicity (*p* < 0.01) [77]. However, the validation study of the NRG/RTOG 0521 trial, which included 202 patients with high-risk PC, did not confirm an association between pretreatment CRP levels and biochemical progression-free survival (adjusted HR 1.07; *p* = 0.60). A negative result on the primary endpoint of this validation study does not invalidate the toxicity observations reported alongside it, which addressed a different question and were analyzed separately. In an unplanned exploratory analysis, higher pretreatment levels of interferon gamma (IFN-γ), interleukin-1 beta (IL-1β), interleukin-2 (IL-2), interleukin-13 (IL-13), and interleukin-23 (IL-23) were each inversely associated with grade ≥ 2 urinary frequency (adjusted OR 0.64, 0.65, 0.71, 0.72, and 0.74, respectively; all *p* < 0.05) [79]. These associations are of considerable interest, since they run counter to the expectation that a more pronounced baseline inflammatory state predisposes to urinary toxicity, and they warrant dedicated investigation. They require cautious interpretation for three reasons. The analysis was exploratory and unplanned, and was conducted across a panel of fifteen cytokines against multiple clinical endpoints; unlike the association between IL-10 and biochemical disease-free survival reported in the same study, the urinary frequency associations were not shown to survive adjustment for multiplicity. The direction of the association is opposite to that reported for IL-6 and TNF-α in the smaller single-center cohorts discussed above, and no mechanism reconciling these observations has been established. The trial population was 90% White, which limits the extent to which these findings can be assumed to hold across more diverse populations. These observations should therefore be regarded as hypothesis-generating and as requiring confirmation in a prospective study with a prespecified analysis plan, as the investigators themselves concluded.

Collectively, these findings indicate that pretreatment CRP is not a robust predictor of biochemical progression-free survival, whereas elevated CRP levels measured during radiotherapy may reflect an acute inflammatory response associated with the development of urinary toxicity. The exploratory cytokine findings from NRG/RTOG 0521 neither support nor exclude a role for baseline immune status in determining urinary toxicity risk; they identify a direction for prospective investigation rather than a predictor available for use.

#### 4.3.5. Systemic Immune-Inflammation Index (SII)

SII, calculated as neutrophil count × platelet count/lymphocyte count, is an emerging biomarker reflecting the balance between the host inflammatory response and immune status. De Bruyn et al. reported, in a prospective study of 32 patients with PC undergoing pelvic radiotherapy, that elevated SII values independently predicted worsening GI symptoms during treatment (*p* = 0.001) [61]. Obesity (*p* = 0.03) and better dietary quality (*p* = 0.015) were identified as protective factors, whereas no significant interaction was observed between SII and either body mass index or dietary quality [61].

The prognostic relevance of SII in PC has subsequently been supported by two recent meta-analyses, showing that elevated SII is significantly associated with poorer overall survival and progression-free survival [80,81]. Nevertheless, evidence supporting the role of SII as a predictor of radiation-induced toxicity remains limited, and larger prospective studies are still needed to confirm its predictive value in this setting.

#### 4.3.6. Radiation-Induced Lymphopenia and Lymphocyte Subpopulations

Pelvic radiotherapy induces a substantial reduction in the absolute lymphocyte count (ALC), which may have important implications for both treatment-related toxicity and tumor control. Pinkawa et al. reported that an early increase in lymphocyte count of ≥0.3 × 10^9^/L at the T2 time point was associated with a lower risk of both late urinary and late bowel toxicity (*p* = 0.02 for both outcomes) [77].

Among lymphocyte subsets, B lymphocytes appear to be the most radiosensitive. D’Auria et al. confirmed that B cells constitute the peripheral blood immune cell population most profoundly affected by radiation exposure [78]. Likewise, Sage et al. reported, in a cohort of 33 patients, that the proportion of B cells declined significantly immediately after radiotherapy—from 10.1% to 6.0% following definitive radiotherapy and from 9.2% to 5.8% following salvage radiotherapy—whereas the proportions of T cells and natural killer (NK) cells remained unchanged immediately after treatment [82]. These observations were further supported by Louagie et al., who found that B lymphocytes represent the most radiosensitive lymphocyte subset in vivo, decreasing to approximately 10% of baseline levels after an equivalent radiation dose of approximately 1.5 Gy [20].

Foro et al. reported, in the largest prospective cohort published to date (214 patients), that in vitro radiation-induced apoptosis of CD4+ T lymphocytes ≤ 28.58% independently predicted an almost twofold higher risk of chronic GU toxicity (HR 1.99; *p* = 0.014). These findings indicate that patients whose CD4+ T lymphocytes are more resistant to radiation-induced apoptosis may have a greater likelihood of developing radiation-induced toxicity [83]. Likewise, Schnarr et al. observed that patients who developed late toxicity exhibited significantly lower levels of lymphocyte apoptosis following exposure to 8 Gy (*p* = 0.01), with a negative predictive value ranging from 95% to 100% [84].

Radiation-induced lymphopenia is substantially more pronounced among patients receiving pelvic nodal irradiation. Al-Hamami et al. reported that lymphopenia (ALC < 1000 cells/μL) was observed in 8% of patients treated with five-fraction SBRT, 17% of those receiving conventionally fractionated radiotherapy (21 fractions), and 84% of patients undergoing conventionally fractionated radiotherapy combined with pelvic nodal irradiation [85]. Basaran et al. identified the inferior pelvic bone marrow V50 as an important dosimetric predictor of post-radiotherapy grade ≥ 2 lymphopenia [86]. Furthermore, Pavarini et al. developed a predictive model for late grade ≥ 2 lymphopenia with an area under the receiver operating characteristic curve (AUC) of 0.87–0.88, incorporating baseline ALC, smoking status, and the lumbosacral bone marrow V24 as predictive variables [87].

#### 4.3.7. Emerging Biomarkers

Recent studies have identified several emerging biomarkers with potential predictive value for radiation-induced toxicity, although these remain at an early stage of clinical validation. Helissey et al. (RABBIO study) reported that baseline serum and urinary concentrations of macrophage colony-stimulating factor (M-CSF) were inversely correlated with patient-reported quality of life during radiotherapy. In addition, polarization of macrophages toward the M2 phenotype increased significantly 12 weeks after treatment completion [88]. Using urinary extracellular vesicle proteomics, Molony et al. identified a 60-protein signature enriched with neutrophil-associated proteins, including myeloperoxidase (MPO), as a potential predictor of late hematuria [89].

#### 4.3.8. Summary Table of Immunological and Inflammatory Biomarkers

The findings demonstrate that inflammatory and immunological biomarkers provide information complementary to clinical and dosimetric predictors by reflecting individual biological susceptibility to radiation-induced toxicity. Among the biomarkers evaluated, IL-6, TGF-β1, and CRP have been associated with various manifestations of GU and GI toxicity, whereas alterations in lymphocyte subpopulations and lymphocyte apoptosis parameters suggest the existence of patient-specific mechanisms of radiosensitivity.

The constraint is more specific than sample size alone. Across the studies summarized in Table 4, biomarker measurement methods, sampling time points, and cut-off definitions differ between cohorts, so the reported associations are not directly comparable, and no consensus threshold exists for any of these markers. Prospective multicenter studies with prespecified assay protocols and independent validation cohorts would be required before any of these biomarkers could contribute to individual risk stratification.

**Table 4:** Immunological and inflammatory biomarkers predictive of GU and GI toxicity.

Biomarker	Associated Toxicity	Acute/Late	Key Findings	References
Elevated IL-6 during radiotherapy	GU (predominantly)	Acute	Independent predictor in multivariable analysis (*n* = 44)	[51,76,77]
Elevated baseline TGF-β1	GU	Late	Significantly higher in patients with grade 2–3 vs. grade 0–1 toxicity	[51,76,78]
Post-radiotherapy changes in TNF-α	GI	Late	Associated with late bowel toxicity (*p* = 0.03; *n* = 91)	[77]
CRP > 5 mg/L during radiotherapy	GU	Acute	Independent predictor of acute urinary toxicity (*p* < 0.01)	[77]
Pretreatment CRP	Biochemical progression-free survival	—	No significant association in NRG/RTOG 0521 (adjusted HR 1.07; *p* = 0.60)	[79]
Elevated SII	GI	Acute	Independently associated with worsening GI symptoms during radiotherapy (*p* = 0.001)	[61]
Early lymphocyte increase ≥ 0.3 × 10^9^/L	GU and GI	Late (protective)	Associated with reduced risk of late GU and GI toxicity (*p* = 0.02 for both)	[77]
B lymphocytes	Most radiosensitive lymphocyte subset	Acute	Declined to approximately 10% of baseline levels after irradiation	[20,82]
CD4+ T-cell apoptosis ≤ 28.58%	GU	Late	HR 1.99 (*p* = 0.014; *n* = 214)	[83,84]
Baseline M-CSF (serum/urine)	GU (quality of life)	Acute	Inversely correlated with FACT-P quality-of-life scores	[88]
Neutrophil-associated proteomic signature	GU (late hematuria)	Late	Sixty-protein signature; MPO identified as a potential predictor	[89]

### 4.4. Genetic and Radiogenomic Predictors

The substantial interindividual variability observed in response to radiotherapy, despite comparable clinical and dosimetric characteristics, has led to the development of radiogenomics. This rapidly advancing field explores how germline genetic variants influence individual susceptibility to radiation-induced toxicity.

#### 4.4.1. Genome-Wide Association Studies (GWASs) from the Radiogenomics Consortium

The largest GWAS meta-analysis reported to date, performed by Kerns et al. within the Radiogenomics Consortium and involving 3871 patients of European ancestry, identified three genome-wide significant loci: rs17055178, associated with rectal bleeding (*p* = 6.2 × 10^−10^); rs10969913, associated with decreased urinary stream (*p* = 2.9 × 10^−10^); and rs11122573, associated with hematuria (*p* = 1.8 × 10^−8^) [18]. The most likely causal variants at these loci were located within regulatory genomic regions, with several predicted to modulate the expression of nearby genes. Previously identified variants were also replicated, including rs17599026, associated with urinary frequency; rs7720298, associated with decreased urinary stream; and rs1801516 in the *ATM* gene, associated with overall radiation toxicity [18]. Notably, two of these variants (rs10969913 and rs17599026) showed comparable effects in Japanese cohorts treated with photon radiotherapy, supporting their potential transethnic applicability [18].

Bouzaki et al. (2025) integrated rectal surface dose maps with genetic data from the REQUITE study (*n* = 1293) and identified the inferior posterior rectum as a critical anatomical region in which genetic variation modifies the dose–toxicity relationship [90]. These findings provide further support for the future development of genetically informed, personalized radiotherapy planning [90].

#### 4.4.2. PROSTOX: A Germline MicroRNA Polymorphism-Based Signature

Candidate-gene studies in radiogenomics have a consistent pattern: individual cohorts report statistically significant associations that subsequent larger studies and meta-analyses have generally failed to replicate. The RAPPER study, which sought independent validation of *ATM*, *TGFB1*, *SOD2*, *XRCC1*, and *XRCC3* variants in large prostate and breast cancer cohorts, could not reproduce most previously reported associations [19]. The findings summarized below should be read against this background: they are individually plausible but collectively unconfirmed, and none currently supports clinical genotyping for toxicity risk.

PROSTOX is a germline genomic signature derived from single-nucleotide polymorphisms (SNPs) that alter microRNA binding sites (mirSNPs) and was developed to estimate the risk of late grade ≥ 2 GU toxicity following prostate radiotherapy. Kishan et al. initially developed predictive models incorporating 22 mirSNPs for conventionally fractionated radiotherapy and 32 mirSNPs for SBRT, with AUC values ranging from 0.76 to 0.87. These models successfully distinguished patients at high risk of toxicity, with rates of 64.7–71.5%, from those at low risk, whose toxicity rates ranged from 3.9% to 7.5% [91].

The PROSTOX signature was subsequently evaluated in a secondary analysis of the MIRAGE trial (NCT04384770), a randomized trial designed to compare MRI-guided with CT-guided SBRT. In this cohort of 148 patients, PROSTOX predicted late grade ≥ 2 GU toxicity with an AUC of 0.76 and correlated with toxicity severity (*p* = 1.2 × 10^−9^) [24]. This analysis was conducted within a trial cohort rather than in an independent validation population, and the signature has not to date been replicated in a cohort external to its development and testing framework.

#### 4.4.3. The GARUDA Trial: Decision-Impact Assessment of PROSTOX

The GARUDA trial (NCT04624256) was a prospective phase II study conducted to assess the clinical impact of PROSTOX testing on treatment selection and treatment-related toxicity. Among the 208 enrolled patients, 86.5% were categorized as low risk and 13.5% as high risk for developing late grade ≥ 2 GU toxicity [92].

The study demonstrated a significant influence of radiogenomic testing on treatment decision-making. Among patients classified as low risk, 98.6% elected to undergo SBRT, whereas 42.9% of high-risk patients chose moderately hypofractionated radiotherapy (MHFRT) instead of SBRT (*p* < 0.001) [92]. In the preliminary analysis after more than 18 months of follow-up, late grade ≥ 2 GU toxicity occurred in 4.8% of patients in the low-risk group, compared with 27.3% in the high-risk group (*p* = 0.015) [93].

At 2 years, the cumulative incidence of late grade ≥ 2 GU toxicity across the whole cohort was 18%, which the investigators reported as lower than anticipated from historical series [93]. Several features of the trial design limit the inferences that can be drawn from this comparison. GARUDA was a single-arm study without a randomized control group, so the observed incidence can only be compared with historical cohorts that differ in radiotherapy technique, dose–volume constraints, image guidance, and toxicity ascertainment. Treatment allocation was determined by patient choice following disclosure of the genomic risk category rather than by protocol, which introduces the possibility that high-risk patients who elected moderately hypofractionated radiotherapy differed systematically from those who did not. A follow-up of 2 years is also short relative to the natural history of late GU toxicity, which can manifest beyond this interval. The GARUDA data therefore establish that PROSTOX testing influences treatment selection, but do not establish that genomically guided selection reduces late GU toxicity; a randomized comparison with longer follow-up would be required to support that conclusion.

#### 4.4.4. Candidate Genes

(a) *TGFB1* (*C-509T* and Leu10Pro)

Mališić et al. reported, in a cohort of 88 patients, that the CT genotype of the *TGFB1* C-509T polymorphism may represent a protective biomarker for acute GU and GI toxicity, whereas the variant of *XRCC3* Thr241Met was associated with a higher risk of acute GU toxicity [94]. Similarly, De Langhe et al. reported, in 322 patients treated with IMRT, that both the −509 CT/TT genotypes (*p* = 0.010) and the codon 10 TC/CC genotypes (*p* = 0.005) were independently associated with an increased risk of radiation-induced acute nocturia [95].

However, a meta-analysis including 2782 participants from 11 cohorts did not confirm an association between rs1800469 (C-509T) and either late fibrosis or overall radiation toxicity (OR 0.98; 95% CI 0.85–1.11) [96]. Likewise, another meta-analysis including 28 studies reported no significant relationship between *TGFB1* polymorphisms and the risk of late radiation toxicity [97]. The weight of evidence therefore does not support an association between *TGFB1* C-509T and radiation toxicity. The positive findings derive from cohorts of 88 and 322 patients, whereas the negative findings come from pooled analyses of 2782 participants across 11 cohorts and from a separate meta-analysis of 28 studies. Where cohort studies and adequately powered meta-analyses diverge in this way, the meta-analytic result is the more reliable estimate.

(b) *RNASEL*

Petrović et al. reported, in a cohort of 81 patients, that the TT genotype of *RNASEL* rs12757998 was significantly associated with the severity of acute GU toxicity, whereas the CT genotype correlated with the severity of late GI toxicity [98]. Furthermore, the CC genotype of rs12757998, together with the AA genotype of rs627928, was identified as independent candidate biomarkers predictive of radiation-induced fatigue [98]. Earlier, Schoenfeld et al. found that the rs12757998 variant was associated with elevated circulating CRP and IL-6 levels, suggesting a biological relationship between *RNASEL*, systemic inflammation, and the response to radiotherapy [99].

Both *RNASEL* findings derive from single cohorts (*n* = 81 and *n* = 1057, respectively), and neither has been independently replicated in prostate radiotherapy populations.

(c) Other Candidate Genes

Guhlich et al. carried out comprehensive genotyping of *TGFB1* and *TGFBR1* (40 polymorphisms) in a cohort of 240 patients and reported that the *TGFBR1* rs10512263 variant was significantly associated with acute grade ≥ 2 toxicity (RR 2.17; 95% CI 1.41–3.31). Moreover, carriers of at least two risk genotypes had an increased likelihood of developing late toxicity [100]. The *TGFBR1* rs10512263 association reported by Guhlich et al. in 240 patients has not been independently replicated [100]. This is consistent with the broader pattern established by the RAPPER study, in which most previously reported candidate-gene associations, including those for *ATM*, *TGFB1*, *SOD2*, *XRCC1*, and *XRCC3*, failed independent validation in large prostate and breast cancer cohorts [19]. Genome-wide approaches with adequate statistical power, such as the Radiogenomics Consortium meta-analysis [18], have proven more reproducible than the candidate-gene paradigm.

#### 4.4.5. Epigenomic and Integrative Approaches

Lopez-Pleguezuelos et al. (2025) performed the first genome-wide epigenome-wide association study (EWAS) in a cohort of 105 patients, identifying 56 differentially methylated pretreatment loci associated with late grade ≥ 2 toxicity [101]. Methylation-based risk scores demonstrated excellent discriminatory performance, with AUCs ranging from 0.87 to 0.98. Gene Ontology analysis additionally identified enrichment of pathways involved in DNA repair, inflammatory responses, and oxidative stress [101].

Franco et al. developed a polygenic risk score model incorporating SNP–SNP interactions (PRSi), demonstrating improved predictive performance over conventional additive polygenic risk scores, with AUCs ranging from 0.61 to 0.78 across different toxicity endpoints [102].

#### 4.4.6. Summary Table of Genetic and Radiogenomic Predictors

However, the clinical implementation of radiogenomics remains limited by the lack of methodological standardization, the relatively small sample sizes of candidate gene studies, challenges related to the reproducibility of reported associations, and the need for prospective external validation. Among radiogenomic biomarkers for GU toxicity, PROSTOX has progressed furthest toward clinical evaluation: it has been assessed within a randomized trial cohort (MIRAGE) and shown to influence treatment selection in a prospective single-arm study (GARUDA). It has not been validated in an independent external cohort, and its effect on toxicity outcomes has not been tested against a randomized control. Both the MIRAGE secondary analysis and the GARUDA cohort were conducted at a single institution, and the ethnic composition of the development and testing populations has not been reported in a manner that allows the transportability of the signature to be assessed. PROSTOX should therefore be regarded as promising but still requiring independent external validation in larger, ethnically diverse cohorts with longer follow-up before any role in routine clinical decision-making can be considered (Table 5).

**Table 5:** Genetic and radiogenomic predictors of GU and GI toxicity following PC radiotherapy.

Genetic/Radiogenomic Factor	Associated Toxicity	Key Findings	Type of Supporting Evidence	References
GWAS: rs17055178	Rectal bleeding	*p* = 6.2 × 10^−10^ (*n* = 3871)	GWAS meta-analysis	[18]
GWAS: rs10969913	Decreased urinary stream	*p* = 2.9 × 10^−10^	GWAS meta-analysis	[18]
GWAS: rs11122573	Hematuria	*p* = 1.8 × 10^−8^	GWAS meta-analysis	[18]
PROSTOX signature (mirSNPs)	Late grade ≥ 2 GU toxicity	AUC 0.76 in MIRAGE (*n* = 148); 27.3% vs. 4.8% late grade ≥ 2 GU toxicity by risk group in GARUDA (*n* = 208)	Secondary analysis within RCT cohort (MIRAGE); single-arm decision-impact study (GARUDA), 2-year follow-up; no independent external validation	[24,91,92,93]
*TGFB1* C-509T polymorphism	Acute GU and GI toxicity; potential protective effect	CT genotype reported as potentially protective; conflicting evidence from negative meta-analyses	Conflicting evidence	[94,95,96,97]
*RNASEL* rs12757998	Acute GU toxicity severity	TT genotype significantly associated with acute GU toxicity severity (*n* = 81)	Single cohort (*n* = 81); not independently replicated	[98,99]
TGFBR1 rs10512263	Acute grade ≥ 2 toxicity	RR 2.17 (*n* = 240)	Single cohort (*n* = 240) with functional validation; not independently replicated	[100]
Pretreatment DNA methylation profile	Late GU and GI toxicity	AUC 0.87–0.98 (*n* = 105)	Exploratory study	[101]
PRSi score incorporating SNP–SNP interactions	Multiple toxicity endpoints	AUC 0.61–0.78 (*n* = 1387)	REQUITE cohort	[102]

Type of supporting evidence refers to the study design(s) from which the association was derived, not a formal grading of evidence quality; predictors evaluated within randomized controlled trials reflect observational associations within the trial cohort unless the predictor itself was the randomized variable.

Integrating genomic information with clinical and dosimetric parameters into multimodal predictive models, including through machine learning approaches, is a plausible direction for individualised risk stratification, but no such model has yet been prospectively validated in an external cohort, and none is currently suitable for routine clinical use.

Table 6 summarizes the methodological heterogeneity identified across the studies cited in Table 1, Table 2, Table 3, Table 4 and Table 5, including the grading instrument, follow-up duration, and fractionation schedule reported for each study.

## 5. Discussion

This narrative review synthesizes the current evidence regarding predictive factors for GU and GI toxicity following modern radiotherapy for PC, focusing on four principal categories of determinants: clinical, dosimetric, immunological/inflammatory, and genetic/radiogenomic factors. Collectively, the available evidence indicates that post-radiotherapy toxicity arises from a complex multifactorial process in which no single predictor is sufficient to accurately estimate individual risk. Rather, susceptibility to treatment-related complications appears to be determined by the interaction of multiple clinical, biological, and treatment-related factors.

### 5.1. Distinct Predictive Profiles for Gu and Gi Toxicity

One of the principal findings of this review is that the predictive factors for GU toxicity differ substantially from those associated with GI toxicity, reflecting distinct underlying pathophysiological mechanisms. GU toxicity is influenced predominantly by pretreatment clinical characteristics—particularly baseline IPSS, prostate volume, previous TURP, and smoking status—whereas bladder dosimetric parameters generally demonstrate a more limited and less consistent predictive value than rectal dosimetric variables [33]. This observation is consistent with the comprehensive review by Martin et al. published in The Lancet Oncology, which concluded that dose–response relationships for the urinary bladder remain less clearly defined than those for the rectum, partly because of substantial interfraction variability in bladder filling and volume [33], a limitation that online adaptive delivery with daily dose accumulation is now being investigated to overcome [74,75].

In contrast, GI toxicity demonstrates a much stronger association with rectal dose–volume parameters—including V70, V60, and, in the setting of SBRT, V38—as well as with specific pretreatment clinical factors such as anticoagulant therapy, cardiovascular disease, and pre-existing anorectal pathology [53,54,55,56,57,60]. These findings suggest that, unlike GU toxicity, the risk of GI toxicity is determined to a greater extent by radiation exposure of anorectal structures together with factors affecting vascular fragility and rectal mucosal integrity.

The recent identification of specific pelvic floor and anorectal substructures—including the iliococcygeus muscle, the levator ani muscle, and the anal sphincter region—as predictors of distinct anorectal complications, such as fecal incontinence and increased stool frequency, represents a significant advance in understanding the mechanisms underlying GI toxicity and provides new opportunities for optimizing radiotherapy treatment planning [59,64,65].

Taken together, these findings support the development of separate predictive models for GU and GI toxicity, rather than relying on a single, global model of radiation-induced toxicity.

### 5.2. Relationship Between Acute and Late Toxicity

The association between acute and late toxicity, quantified in Section 3, is well established and underlies the consequential late toxicity model rather than constituting a novel finding of this review [17]. Its contribution here is practical rather than conceptual: acute toxicity is the only predictor in this review that becomes available during treatment rather than before it. Whereas baseline IPSS, prostate volume, and dosimetric parameters inform decisions made at planning, the emergence of grade ≥ 2 acute toxicity identifies a higher-risk subgroup at a point where surveillance intensity, symptomatic management, and follow-up scheduling can still be adjusted. This distinguishes it operationally from the other predictors discussed, irrespective of its effect size. Whether this window can be used therapeutically, for instance, by triggering earlier initiation of symptom-directed management or closer surveillance protocols in patients who develop grade ≥ 2 acute toxicity, has not been tested in randomized data and represents a specific, testable question for future prospective trials.

Conditional risk analysis from the RTOG 0126 trial (*n* = 1499; median follow-up, 8.4 years) further showed that the probability of late toxicity progressively declined over time. The estimated probability of developing grade ≥ 2 GU toxicity during the following five years decreased from 17.89% at study enrollment to 3.02% among patients who remained free of toxicity for five years. Likewise, the corresponding probability of grade ≥ 2 GI toxicity fell from 9.57% to 1.54% [31]. These findings indicate that most late toxicities occur within the first few years after treatment and that patients who remain toxicity-free throughout this interval have a relatively low residual risk of subsequent complications.

The COBALT study, another IPD meta-analysis from the MARCAP Consortium (*n* = 6761), identified an inverse relationship between late grade ≥ 2 GI toxicity and biochemical recurrence (subdistribution HR [sHR] 0.64; *p* = 0.03), suggesting that shared biological and dosimetric mechanisms may simultaneously influence rectal toxicity and local tumor control. One hypothesis proposed by the investigators is that anterosuperior displacement of the prostate during treatment may lead to both increased rectal radiation exposure and improved dose coverage of dominant posterior intraprostatic lesions.

### 5.3. Impact of Follow-Up Duration and Methodological Heterogeneity

The methodological heterogeneity documented in Section 2.3 and Table 6 has a direct consequence for the interpretation of this review: reported incidence figures for the same toxicity endpoint are not comparable across studies using different instruments. The magnitudes summarized in Table 1, Table 2, Table 3, Table 4 and Table 5 should therefore be read as indicating direction and approximate strength rather than as values that could be pooled or transferred between populations.

### 5.4. Emergence of Immunological Biomarkers and Radiogenomics

The evidence reviewed in this study highlights a paradigm shift toward multifactorial predictive models that integrate biological biomarkers with traditional clinical and dosimetric risk factors. Pro-inflammatory cytokines, including IL-6, TNF-α, and TGF-β1, the systemic immune-inflammation index (SII), and dynamic changes in lymphocyte subpopulations during radiotherapy have all been associated with GU and GI toxicity in cohort studies. These associations derive from cohorts of 32 to 214 patients, all single-center, none externally validated, and with heterogeneous assay methods and sampling schedules [20,51,61,76,77,78,79,82,83,84,85,86,87]. At present, they describe biologically plausible mechanisms rather than clinically actionable predictors.

One particularly noteworthy finding is that B lymphocytes represent the most radiosensitive lymphocyte subset in peripheral blood. Furthermore, in vitro radiation-induced apoptosis of CD4+ T lymphocytes ≤ 28.58% was linked to an almost twofold higher risk of chronic GU toxicity (HR 1.99; *p* = 0.014), indicating that patients whose T lymphocytes are more resistant to radiation-induced apoptosis may be at increased risk of treatment-related toxicity [83,84]. Functional lymphocyte assays are therefore a plausible avenue for predicting individual radiosensitivity. The supporting evidence comes from a single cohort of 214 patients without independent replication, and the assay itself has not been standardized across laboratories, both of which would need to be addressed before clinical evaluation.

Within radiogenomics, PROSTOX is the signature that has advanced furthest toward clinical assessment, having been evaluated within the MIRAGE trial cohort (AUC 0.76) and having demonstrably influenced treatment selection in GARUDA. Both findings are early-phase: the MIRAGE analysis was secondary to a trial with a different primary objective, and GARUDA was single-arm with 2-year follow-up [24,91,92,93]. At 2 years, the cumulative incidence of late grade ≥ 2 GU toxicity across the GARUDA cohort was 18%, lower than reported in historical series; because the trial had no randomized control arm, this comparison indicates that genomically guided selection altered treatment choice for a substantial proportion of high-risk patients, but does not establish that it reduced toxicity relative to standard care. Notably, 42.9% of patients categorized as high risk by PROSTOX elected to receive moderately hypofractionated radiotherapy rather than SBRT [92,93]. Moreover, the Radiogenomics Consortium GWAS meta-analysis (*n* = 3871) identified genome-wide significant loci associated with rectal bleeding, decreased urinary stream, and hematuria, providing further evidence that genetic variation contributes to susceptibility to radiation-induced toxicity [18].

Nevertheless, the RAPPER study highlighted the difficulties in reproducing previously reported associations involving candidate genes, including *ATM*, *TGFB1*, *SOD2*, *XRCC1*, and *XRCC3*, across independent cohorts, underscoring the persistent challenge of achieving robust validation in radiogenomic research [19].

### 5.5. Integrated Predictive Models

The development of integrated predictive models has become one of the leading research priorities in contemporary PC radiotherapy. Conventional normal tissue complication probability (NTCP) models, which rely primarily on dosimetric parameters, have shown moderate predictive performance for radiation-induced toxicity and have laid the groundwork for individualized risk assessment strategies [58,60].

The findings presented in this review indicate that combining clinical, dosimetric, immunological, and genetic variables may substantially enhance predictive performance compared with models based on a single category of predictors. In particular, integrating pretreatment clinical characteristics, dose–volume parameters, inflammatory biomarkers, and radiogenomic information may facilitate the development of more accurate and personalized prediction models tailored to each patient’s biological and clinical profile [20,46,47,48,49,50,51,52,53,54,55,56,57,58,59,60,61,62,63,64,65,66,67,68,69,70,71,72,73,74,75,76,77,79,80,81,82,83,84,85,86,87,88,89,90,91,92,93,94,95,96,97,98].

Despite these advances, most predictive models currently available have been derived from relatively small and heterogeneous patient populations and have undergone only limited external validation. Methodological standardization, prospective multicenter validation, and demonstration of clinical utility remain essential prerequisites before these tools can be routinely implemented in clinical practice. Ultimately, integrating clinical, dosimetric, imaging, and biological information into multimodal predictive models represents one of the most promising strategies for advancing precision medicine in radiation oncology [63,64,65,66,67,68,69,70,71,72,73,74,75,76,77].

### 5.6. Clinical Implications

The predictors reviewed here differ substantially in the strength of evidence supporting them, and this difference should govern how each is used. The recommendations below are grouped accordingly.

Supported by randomized or prospectively validated evidence, and appropriate for routine use. Baseline IPSS should be documented before treatment in every patient, since it is the most consistently replicated predictor of GU toxicity across trial and cohort data and is the only clinical variable that independently predicted late GU toxicity in more than one of the cohorts reviewed. A history of TURP should be recorded and factored into technique selection. Rectal dose–volume constraints derived from randomized data should be applied as planning objectives. Anticoagulant and antiplatelet therapy should be documented, given the consistency of its association with late rectal bleeding across cohorts.

Supported by cohort evidence with external validation, and reasonable to apply where feasible: Where planning systems and contouring workflows permit, dose to the anorectal substructures and to the urethra can be constrained using published thresholds; the supporting NTCP models have been externally validated, though not tested prospectively as planning objectives. Smoking status should be recorded, and the association with late GU toxicity provides an additional argument for cessation support, independent of its oncological rationale.

Not yet appropriate for clinical decision-making: Bladder dose–volume constraints, including the QUANTEC recommendations, rest on a weaker evidence base than their rectal counterparts and are limited by interfraction variation in bladder filling; they should be regarded as planning aims rather than validated predictors. Accumulated-dose analyses on adaptive MR-guided platforms may eventually support validated bladder constraints, but no such constraint is yet available for clinical use. Bladder substructure constraints derive from single-institution modelling and are not yet established. Inflammatory and immunological biomarkers have no established assay thresholds and should not inform individual decisions. PROSTOX has been evaluated within a trial cohort and shown to influence treatment selection, but has not been validated externally and has not been shown in a randomized comparison to reduce toxicity; its use outside research protocols is not currently supported.

A note on what is not actionable: Age, diabetes, and BMI appear as statistically significant predictors in individual cohorts but are confounded with other variables in ways that current analyses do not resolve (Section 4.1.2 and Section 4.1.3). None of the three should be used as a standalone criterion for modifying treatment. In particular, chronological age alone should not preclude hypofractionation in otherwise suitable patients, and higher BMI should not be interpreted as protective in patient counselling.

Monitoring during treatment: Grade ≥ 2 acute toxicity identifies patients at elevated risk of late toxicity and, unlike the pretreatment predictors, becomes available while management can still be adjusted. It is a reasonable trigger for closer follow-up, though no intervention has been shown in randomized data to modify late toxicity risk once acute toxicity has occurred. Prospective trials evaluating acute toxicity as an enrollment trigger for early prophylactic intervention, such as targeted anti-inflammatory or symptom-directed protocols initiated at first-grade ≥ 2 event, would clarify whether this operational window can be translated into a modifiable outcome.

## 6. Limitations

This narrative review is subject to several methodological limitations that should be considered when interpreting the findings.

First, as a narrative review, this work does not provide the exhaustive study retrieval and quantitative synthesis of a systematic review with meta-analysis. Selection of included studies was guided by predefined eligibility criteria and a structured search process (documented in Section 2 and Supplementary Table S1), which reduces but does not eliminate selection bias. Readers should interpret the summarized associations in light of the variability in study quality across the included evidence, which is made explicit through study design classification in tables and through qualified language in the text for preliminary or exploratory findings.

Second, considerable methodological heterogeneity was observed across the included studies, including differences in radiotherapy techniques (3D-CRT, IMRT, VMAT, and SBRT), evaluated dosimetric parameters, toxicity assessment methods (RTOG, CTCAE, and patient-reported outcomes [PROs]), and duration of follow-up. The PACE-B trial showed that the choice of toxicity grading system (RTOG versus CTCAE) may substantially affect the reported incidence of treatment-related toxicity, thereby limiting direct comparisons across studies [30].

Third, evidence regarding immunological and inflammatory biomarkers is derived predominantly from relatively small cohorts (32–91 patients), with limited standardization of biomarker assessment and longitudinal monitoring. The absence of external validation and standardized cytokine quantification methods limits the incorporation of these biomarkers into robust predictive models and reduces the comparability of findings across studies.

Fourth, within the field of radiogenomics, most candidate gene associations have not been successfully replicated in independent cohorts, and the RAPPER study highlighted the considerable challenges associated with validating previously reported genetic associations [19]. PROSTOX currently has the most supporting evidence of any radiogenomic signature for GU toxicity, though this evidence derives primarily from a single trial cohort (MIRAGE) and one single-arm decision-impact study (GARUDA), neither of which constitutes external validation. Evidence regarding its performance across ethnically diverse populations remains limited.

Fifth, limiting the literature search to English-language publications may have introduced language bias. Furthermore, potential publication bias should also be considered, since studies reporting positive findings are generally more likely to be published than those describing negative or inconclusive results.

Sixth, differences in the definition and reporting of late toxicity, particularly regarding clinician-reported versus patient-reported outcomes, may have affected the consistency and comparability of findings across studies [30].

Overall, these limitations highlight the need for large, prospective, multicenter studies using standardized methodologies, uniform toxicity assessment instruments, and long-term follow-up (≥5 years) to facilitate external validation and support the clinical implementation of the predictive factors identified to date.

## 7. Conclusions

GU and GI toxicity associated with PC radiotherapy is multifactorial and results from the complex interplay of clinical, dosimetric, immunological, and genetic determinants.

The evidence reviewed indicates that these two categories of toxicity are characterized by distinct predictive profiles. GU toxicity is influenced predominantly by pretreatment clinical characteristics, including baseline urinary symptom severity, prostate volume, and previous prostate interventions, whereas GI toxicity is more closely associated with rectal dosimetric parameters, as well as with specific comorbidities and concomitant treatments.

Dosimetric parameters remain fundamental components of toxicity risk assessment, while the evaluation of individual anatomical substructures of the urinary bladder and rectum provides additional opportunities to optimize radiotherapy treatment planning. Furthermore, the development of acute toxicity represents an important predictor of subsequent late complications, emphasizing the importance of careful clinical monitoring from the earliest stages of treatment.

Immunological and radiogenomic biomarkers are promising avenues for treatment personalization, but none is currently ready for clinical application. PROSTOX is the radiogenomic signature with the most supporting evidence for late GU toxicity; it remains promising but still requires independent external validation in larger, ethnically diverse cohorts with longer follow-up, and its use outside research protocols is not presently supported. Inflammatory biomarkers have no established assay thresholds and should not inform individual treatment decisions.

Overall, integrating clinical, dosimetric, and biological variables into multimodal predictive models has the potential to improve individualized risk stratification and support the implementation of precision radiotherapy in patients with PC. Large prospective multicenter studies using standardized methodologies and adequate long-term follow-up remain necessary to validate such predictive models and facilitate their translation into routine clinical practice.

## Figures and Tables

**Table 6:** Sources of methodological heterogeneity across studies included in the narrative synthesis.

Dimension	Variants Identified	Representative Examples
Clinician-reported grading instrument	RTOG; RTOG/EORTC; NCI CTC v2.0; CTCAE v3.0–v5.0 (version unspecified in several reports); LENT/SOMA; Vienna Rectoscopy Score; Jorge & Wexner score; ALC-based grading; QUANTEC (dose-constraint framework); institution- or study-specific scales; administrative ICD-10 coding	RTOG [29,68,91]; CTCAE [30,35,44,59,62,100]; LENT/SOMA [58,100]; Vienna Rectoscopy Score [57]; Jorge & Wexner [67]; institution-specific [56,65]
Concurrent use of more than one instrument within a single study	Several studies apply different instruments to acute versus late endpoints, or report clinician-graded and patient-reported outcomes in parallel	CTCAE (acute) and RTOG/EORTC or LENT-SOMA (late) in the same cohort [32,62,100]; RTOG and CTCAE reported in parallel [22,23]; CTCAE compared directly with RTOG [24]
Patient-reported outcome instruments used as primary or co-primary endpoint	IPSS; EPIC/EPIC-26; PCSS; FACT-P; EORTC QLQ-PRT20	IPSS as the primary endpoint (≥10-point increase) [69]; EPIC only, without a clinician-graded scale [41,77]; FACT-P and IPSS in parallel [88]; EORTC QLQ-PRT20 [61]
Definition of acute toxicity	Same-week to 90-day windows	≤90 days [25,39]; ≤12 weeks [22]; weekly during treatment [85]
Definition of late toxicity	>90 days to fixed 2-, 3-, or 5-year cumulative incidence	>90 days [25]; 2-year endpoint [19,35,102]; 3-year cumulative incidence [34,58]; 5-year cumulative incidence [57]
Median follow-up duration	Acute-only reporting to 10.7 years	Early/acute only [22]; 22–30 months [21,38]; 44–48 months [36,54]; 72–74 months [17,23]; 8.4 years [25,31]; 10.6–10.7 years [29]
Fractionation schedule	Conventional (1.8–2.0 Gy/fraction) to ultra-hypofractionation/SBRT (≥5 Gy/fraction)	68–78 Gy conventional [32]; 70.2–79.2 Gy/39–44 fr [25,31]; 74/60/57 Gy moderate hypofractionation [13]; 42.7 Gy/7 fr and 36.25 Gy/5 fr [22,29,35,44]
Endpoint basis	Clinician-graded only; patient-reported only; both in parallel; administrative coding; dose-constraint concordance rather than a graded scale	Clinician-graded only [13,31,32]; patient-reported only [41,69]; both in parallel [35,44,100]; QUANTEC constraints used as a reference framework rather than a grading instrument [73]

Note: This table summarizes heterogeneity identified through structured verification of the methodological characteristics of studies cited in Table 1, Table 2, Table 3, Table 4 and Table 5, covering all primary studies cited in these tables. For approximately one-quarter of these studies, the grading instrument was not stated explicitly in the sources accessed; these are marked “not explicit” in the tables rather than inferred.

## Data Availability

The original contributions presented in this study are included in the article/Supplementary Material. Further inquiries can be directed to the corresponding authors.

## Supplementary Materials

The following supporting information can be downloaded at: https://www.mdpi.com/article/10.3390/life16091477/s1, Table S1. Search strategy and results by database and topic; Text S1.1. Clinical predictors (Search ID S1); Text S1.2. Dosimetric parameters (Search ID S2); Text S1.3. Immunological/inflammatory biomarkers (Search ID S3); Text S1.4. Radiogenomics/genetic factors (Search ID S4).

## Abbreviations

3D-CRT (Three-Dimensional Conformal Radiotherapy)	A radiotherapy technique that uses three-dimensional imaging to conform the radiation dose to the target volume.
ADT (Androgen Deprivation Therapy)	Hormonal therapy commonly combined with radiotherapy in the treatment of PC.
ALC (Absolute Lymphocyte Count)	Absolute peripheral blood lymphocyte count; treatment-related lymphopenia has been associated with radiation-induced toxicity and clinical outcomes.
AUC (Area Under the Receiver Operating Characteristic Curve)	Measure of predictive model performance; values closer to 1 indicate better discriminative ability.
BOO (Bladder Outlet Obstruction)	Bladder outlet obstruction, commonly associated with benign prostatic hyperplasia and recognized as a predictor of GU toxicity.
CART (Classification and Regression Tree)	Machine learning algorithm used for predictive modeling.
CBCT (Cone-Beam Computed Tomography)	Cone-beam computed tomography used for image-guided radiotherapy (IGRT).
CI (Confidence Interval)	Statistical confidence interval, typically reported as a 95% confidence interval.
CRP (C-Reactive Protein)	Nonspecific biomarker of systemic inflammation investigated as a predictor of radiation-induced toxicity.
CRT (Conventional Radiotherapy)	Conventionally fractionated radiotherapy.
CTCAE (Common Terminology Criteria for Adverse Events)	Standardized grading system for adverse events and treatment-related toxicity.
CTV (Clinical Target Volume)	Tissue volume encompassing the gross tumor and areas at risk of microscopic disease.
D_0.1cc_, D_1cc_, D_10cc_	Radiation dose delivered to 0.1 cm^3^, 1 cm^3^, and 10 cm^3^ of tissue, respectively.
Dmax (Maximum Dose)	Maximum dose delivered to an organ or specified tissue volume.
Dmean (Mean Dose)	Mean radiation dose delivered to an organ or target volume.
EBRT (External Beam Radiation Therapy)	External beam radiotherapy.
EPIC (Expanded PC Index Composite)	Validated patient-reported outcome questionnaire assessing urinary, bowel, sexual, and hormonal quality of life in patients with PC.
EWAS (Epigenome-Wide Association Study)	Genome-wide epigenetic association study used to identify differentially methylated loci associated with clinical phenotypes.
FACT-P (Functional Assessment of Cancer Therapy—Prostate)	Validated PC-specific quality-of-life questionnaire.
GARUDA	Prospective phase II clinical trial evaluating the impact of the PROSTOX radiogenomic signature on treatment selection and late urinary toxicity after PC radiotherapy.
GI (Gastrointestinal)	Referring to GI toxicity, primarily involving the rectum, distal colon, and anal sphincter.
GU (GU)	Referring to GU toxicity involving the urinary bladder, urethra, bladder trigone, and other lower urinary tract structures.
GWAS (Genome-Wide Association Study)	A genome-wide association study used to identify genetic variants associated with clinical phenotypes.
HF (Hypofractionation)	Delivery of larger radiation doses per fraction over a reduced number of treatment sessions.
HR (Hazard Ratio)	Measure of relative risk commonly used in time-to-event analyses.
IGRT (Image-Guided Radiation Therapy)	Radiotherapy using imaging guidance to verify patient positioning before or during treatment.
IL-2 (Interleukin-2)	Cytokine involved in T-cell activation and proliferation; investigated as a potential immunological biomarker.
IL-6 (Interleukin-6)	Pro-inflammatory cytokine implicated in radiation-induced inflammatory responses and investigated as a biomarker of GU and GI toxicity following PC radiotherapy.
BMI (Body Mass Index)	Ratio of body weight (kg) to height squared (m^2^); higher BMI has paradoxically been associated with a reduced risk of rectal bleeding in some studies.
IMRT (Intensity-Modulated Radiation Therapy)	Advanced radiotherapy technique that modulates beam intensity to optimize dose distribution while sparing organs at risk.
IPD (Individual Patient Data)	Individual-level patient data used in meta-analyses to improve analytical precision.
IPSS (International Prostate Symptom Score)	Validated questionnaire assessing lower urinary tract symptoms; a major predictor of GU toxicity.
LASSO (Least Absolute Shrinkage and Selection Operator)	A penalized regression method widely used for variable selection and predictive modeling.
MARCAP (Meta-Analysis of Randomized Trials in Cancer of the Prostate)	International consortium performing individual patient data meta-analyses of randomized PC trials.
M-CSF (Macrophage Colony-Stimulating Factor)	Emerging biomarker associated with quality of life during radiotherapy.
MHFRT (Moderately Hypofractionated Radiation Therapy)	Moderately hypofractionated radiotherapy, typically delivering 2.4–3.4 Gy per fraction.
MIRAGE (Magnetic Resonance Imaging-Guided SBRT Versus Computed Tomography-Guided SBRT)	Randomized clinical trial comparing MRI-guided and CT-guided SBRT, which also provided the prospective validation cohort for the PROSTOX signature.
mirSNP (microRNA-binding Site Single-Nucleotide Polymorphism)	Single-nucleotide polymorphism that disrupts a microRNA-binding site; forms the basis of the PROSTOX radiogenomic signature.
MPO (Myeloperoxidase)	Neutrophil-derived enzyme identified as a potential biomarker for predicting late hematuria.
MR-guided RT (Magnetic Resonance-Guided Radiation Therapy)	Radiotherapy delivered under magnetic resonance imaging guidance.
NK cells (Natural Killer cells)	Lymphocyte subset involved in innate immune responses.
NTCP (Normal Tissue Complication Probability)	Mathematical model used to estimate the probability of radiation-induced normal tissue complications.
OR (Odds Ratio)	A measure of association expressing the odds of an event occurring in one group relative to another.
PC (Prostate Cancer)	
PRO (Patient-Reported Outcome)	Patient-completed assessments of treatment-related toxicity and health-related quality of life.
PRS/PRSi (Polygenic Risk Score/Polygenic Risk Score with Interactions)	Genetic risk score based on multiple variants; PRSi additionally incorporates SNP–SNP interactions.
PROSTOX	Germline radiogenomic signature based on microRNA-binding site polymorphisms (mirSNPs), developed to predict the risk of late grade ≥ 2 GU toxicity following PC radiotherapy.
PSA (Prostate-Specific Antigen)	Biomarker used for the diagnosis, risk stratification, and monitoring of PC.
PTV (Planning Target Volume)	Target volume that includes the clinical target volume (CTV) plus margins accounting for setup uncertainties and internal organ motion.
*p*-value	Probability that an observed result occurred by chance; values < 0.05 are generally considered statistically significant.
QUANTEC (Quantitative Analyses of Normal Tissue Effects in the Clinic)	Evidence-based guidelines describing radiation dose–response relationships for normal tissues.
*RNASEL* (*Ribonuclease L*)	Gene involved in the innate immune response; its polymorphisms have been associated with the severity of radiation-induced GU and GI toxicity.
ROC (Receiver Operating Characteristic)	Statistical method used to evaluate the discriminatory performance of predictive models.
RR (Relative Risk/Risk Ratio)	Ratio of the incidence of an event in the exposed group compared with the unexposed group.
RT (Radiotherapy)	Radiation therapy.
RTOG (Radiation Therapy Oncology Group)	Standardized toxicity grading system widely used in radiation oncology; also refers to the RTOG cooperative research organization.
SBRT (Stereotactic Body Radiation Therapy)	Highly conformal radiotherapy technique delivering high doses per fraction over a limited number of treatment sessions, typically five fractions.
SII (Systemic Immune-Inflammation Index)	Composite biomarker calculated as *neutrophil count × platelet count/lymphocyte count*; an emerging indicator associated with worsening GI symptoms during radiotherapy.
SNP (Single-Nucleotide Polymorphism)	Genetic variation involving a single nucleotide within the DNA sequence.
TGF-β1 (Transforming Growth Factor Beta 1)	Cytokine involved in tissue fibrosis and remodeling, implicated in the development of late radiation-induced toxicity.
TNF-α (Tumor Necrosis Factor Alpha)	Pro-inflammatory cytokine involved in radiation-induced inflammatory responses and investigated as a potential predictor of GI toxicity.
TURP (Transurethral Resection of the Prostate)	Surgical procedure for benign prostatic obstruction; a history of TURP is associated with an increased risk of late GU toxicity following radiotherapy.
VMAT (Volumetric Modulated Arc Therapy)	Advanced radiotherapy technique delivering radiation through continuous gantry rotation with dynamic modulation of beam intensity and dose rate.
Vx/VxGy (Volume Receiving x Gy)	Percentage or absolute volume of an organ receiving at least *x* Gy of radiation (e.g., V70 denotes the volume receiving ≥ 70 Gy).
