# Supplementary material for: Predictive Factors for Acute and Late Genitourinary and Gastrointestinal Toxicity Following Modern Radiotherapy for Prostate Cancer: A Narrative Review of Clinical, Dosimetric, Immunological, and Genetic Determinants
Source: Life (Basel). 2026 Sep 3;16(9):1477. doi: 10.3390/life16091477 (PMC13608368; doi:10.3390/life16091477)
Supplement: Supplementary file 1 [file life-16-01477-s001.zip › life-4492886-supplementary.pdf]

Supplementary Materials

Predictive Factors for Acute and Late Genitourinary and Gastrointestinal Toxicity Following Modern Radiotherapy for Prostate Cancer

Table S1. Search strategy and results by database and topic.

Search ID	Topic	Database	Date of search	Records retrieved
S1	Clinical predictors	PubMed/MEDLINE	Jan–Mar 2026; reconstructed Aug 2026	923
S1	Clinical predictors	Scopus	Aug 2026	1,611
S1	Clinical predictors	Europe PMC	Aug 2026	331
S2	Dosimetric parameters	PubMed/MEDLINE	Jan–Mar 2026; reconstructed Aug 2026	676
S2	Dosimetric parameters	Scopus	Aug 2026	744
S2	Dosimetric parameters	Europe PMC	Aug 2026	260
S3	Immunological/inflammatory biomarkers	PubMed/MEDLINE	Jan–Mar 2026; reconstructed Aug 2026	54
S3	Immunological/inflammatory biomarkers	Scopus	Aug 2026	92
S3	Immunological/inflammatory biomarkers	Europe PMC	Aug 2026	20
S4	Radiogenomics/genetic factors	PubMed/MEDLINE	Jan–Mar 2026; reconstructed Aug 2026	73
S4	Radiogenomics/genetic factors	Scopus	Aug 2026	94
S4	Radiogenomics/genetic factors	Europe PMC	Aug 2026	29
			**Total (before deduplication)**	**4,907**

Note. Complete verbatim search strings for each database and topic are provided in Supplementary Text S1.1–S1.4 below.

Supplementary Text Verbatim search strings by database and topic.

Boolean operators AND, OR, and NOT are shown as applied in each database's native syntax. Field tags: [MeSH] = Medical Subject Heading (PubMed/MEDLINE); [tiab] = title/abstract field (PubMed/MEDLINE); TITLE-ABS-KEY = title, abstract, and keyword field (Scopus); TITLE_ABS = title/abstract field (Europe PMC).

Text S1.1. Clinical predictors (Search ID S1).

PubMed/MEDLINE:

```
("Prostatic Neoplasms"[MeSH] OR "prostate cancer"[tiab] OR "prostatic neoplasm*"[tiab])
AND ("Radiotherapy"[MeSH] OR "Radiotherapy, Intensity-Modulated"[MeSH] OR "Radiosurgery"[MeSH]
OR "radiotherapy"[tiab] OR "radiation therapy"[tiab] OR "external beam radiotherapy"[tiab]
OR "EBRT"[tiab] OR "IMRT"[tiab] OR "intensity-modulated radiotherapy"[tiab] OR "VMAT"[tiab]
OR "volumetric modulated arc therapy"[tiab] OR "SBRT"[tiab] OR "stereotactic body
radiotherapy"[tiab])
AND ("Radiation Injuries"[MeSH] OR "genitourinary toxicity"[tiab] OR "urinary toxicity"[tiab]
OR "gastrointestinal toxicity"[tiab] OR "rectal toxicity"[tiab] OR "GU toxicity"[tiab]
OR "GI toxicity"[tiab] OR "radiation-induced toxicity"[tiab] OR "treatment-related
toxicity"[tiab]
OR "adverse event*"[tiab])
AND ("predictive factor*"[tiab] OR "risk factor*"[tiab] OR "predictor*"[tiab]
OR "International Prostate Symptom Score"[tiab] OR "IPSS"[tiab] OR "prostate volume"[tiab]
OR "TURP"[tiab] OR "smoking"[tiab] OR "diabetes mellitus"[tiab] OR "anticoagulant*"[tiab]
OR "alpha-blocker*"[tiab] OR "body mass index"[tiab] OR "BMI"[tiab] OR "hemorrhoid*"[tiab]
OR "cardiovascular"[tiab])
NOT (child*[tiab] OR pediatric[tiab] OR paediatric[tiab])
AND ("2000"[PDAT] : "2026"[PDAT]) AND English[la]
```

Scopus:

```
TITLE-ABS-KEY(("prostatic neoplasms" OR "prostate cancer" OR "prostatic neoplasm*")
AND ("radiotherapy" OR "radiation therapy" OR "external beam radiotherapy" OR "EBRT"
OR "IMRT" OR "intensity-modulated radiotherapy" OR "VMAT" OR "volumetric modulated arc
therapy"
OR "SBRT" OR "stereotactic body radiotherapy"))
AND TITLE-ABS-KEY(("genitourinary toxicity" OR "urinary toxicity" OR "gastrointestinal
toxicity"
OR "rectal toxicity" OR "GU toxicity" OR "GI toxicity" OR "radiation-induced toxicity"
OR "treatment-related toxicity" OR "adverse event*")
AND ("predictive factor*" OR "risk factor*" OR "predictor*" OR "International Prostate Symptom
Score"
OR "IPSS" OR "prostate volume" OR "TURP" OR "smoking" OR "diabetes mellitus" OR
"anticoagulant*"
OR "alpha-blocker*" OR "body mass index" OR "BMI" OR "hemorrhoid*" OR "cardiovascular"))
AND NOT TITLE-ABS-KEY(child* OR pediatric OR paediatric)
AND PUBYEAR > 1999 AND PUBYEAR < 2027 AND LANGUAGE(english)
```

Europe PMC:

```
TITLE_ABS:("prostatic neoplasms" OR "prostate cancer" OR "prostatic neoplasm*")
AND TITLE_ABS:("radiotherapy" OR "radiation therapy" OR "external beam radiotherapy" OR "EBRT"
OR "IMRT" OR "intensity-modulated radiotherapy" OR "VMAT" OR "volumetric modulated arc
therapy"
OR "SBRT" OR "stereotactic body radiotherapy")
AND TITLE_ABS:("genitourinary toxicity" OR "urinary toxicity" OR "gastrointestinal toxicity"
OR "rectal toxicity" OR "GU toxicity" OR "GI toxicity" OR "radiation-induced toxicity"
OR "treatment-related toxicity" OR "adverse event*")
AND TITLE_ABS:("predictive factor*" OR "risk factor*" OR "predictor*"
OR "International Prostate Symptom Score" OR "IPSS" OR "prostate volume" OR "TURP" OR
"smoking"
OR "diabetes mellitus" OR "anticoagulant*" OR "alpha-blocker*" OR "body mass index" OR "BMI"
OR "hemorrhoid*" OR "cardiovascular")
NOT TITLE_ABS:(child* OR pediatric OR paediatric)
AND PUB_YEAR:[2000 TO 2026] AND LANG:"eng"
```

Text S1.2. Dosimetric parameters (Search ID S2).

PubMed/MEDLINE:

```
("Prostatic Neoplasms"[MeSH] OR "prostate cancer"[tiab] OR "prostatic neoplasm*"[tiab])
AND ("Radiotherapy"[MeSH] OR "Radiotherapy, Intensity-Modulated"[MeSH] OR "Radiosurgery"[MeSH]
OR "radiotherapy"[tiab] OR "radiation therapy"[tiab] OR "external beam radiotherapy"[tiab]
OR "EBRT"[tiab] OR "IMRT"[tiab] OR "intensity-modulated radiotherapy"[tiab] OR "VMAT"[tiab]
OR "volumetric modulated arc therapy"[tiab] OR "SBRT"[tiab] OR "stereotactic body
radiotherapy"[tiab])
AND ("Radiation Injuries"[MeSH] OR "genitourinary toxicity"[tiab] OR "urinary toxicity"[tiab]
OR "gastrointestinal toxicity"[tiab] OR "rectal toxicity"[tiab] OR "radiation-induced
toxicity"[tiab])
AND ("dose-volume histogram"[tiab] OR "dosimetric parameter*"[tiab] OR "dose-volume"[tiab]
OR "normal tissue complication probability"[tiab] OR "NTCP"[tiab] OR "organ* at risk"[tiab]
OR "OAR"[tiab] OR "dose constraint*"[tiab] OR "rectal dose"[tiab] OR "bladder dose"[tiab]
OR "urethral dose"[tiab] OR "bladder trigone"[tiab] OR "anal sphincter"[tiab] OR "anal
canal"[tiab])
NOT (child*[tiab] OR pediatric[tiab] OR paediatric[tiab])
AND ("2000"[PDAT] : "2026"[PDAT]) AND English[la]
```

Scopus:

```
TITLE-ABS-KEY(("prostatic neoplasms" OR "prostate cancer" OR "prostatic neoplasm*")
AND ("radiotherapy" OR "radiation therapy" OR "external beam radiotherapy" OR "EBRT"
OR "IMRT" OR "intensity-modulated radiotherapy" OR "VMAT" OR "volumetric modulated arc
therapy"
OR "SBRT" OR "stereotactic body radiotherapy"))
AND TITLE-ABS-KEY(("genitourinary toxicity" OR "urinary toxicity" OR "gastrointestinal
toxicity"
OR "rectal toxicity" OR "radiation-induced toxicity")
AND ("dose-volume histogram" OR "dosimetric parameter*" OR "dose-volume"
OR "normal tissue complication probability" OR "NTCP" OR "organ* at risk"
OR "OAR" OR "dose constraint*" OR "rectal dose" OR "bladder dose"
OR "urethral dose" OR "bladder trigone" OR "anal sphincter" OR "anal canal"))
AND NOT TITLE-ABS-KEY(child* OR pediatric OR paediatric)
AND PUBYEAR > 1999 AND PUBYEAR < 2027 AND LANGUAGE(english)
```

Europe PMC:

```
TITLE_ABS:("prostatic neoplasms" OR "prostate cancer" OR "prostatic neoplasm*")
AND TITLE_ABS:("radiotherapy" OR "radiation therapy" OR "external beam radiotherapy" OR "EBRT"
OR "IMRT" OR "intensity-modulated radiotherapy" OR "VMAT" OR "volumetric modulated arc
therapy"
OR "SBRT" OR "stereotactic body radiotherapy")
AND TITLE_ABS:("genitourinary toxicity" OR "urinary toxicity" OR "gastrointestinal toxicity"
OR "rectal toxicity" OR "radiation-induced toxicity")
AND TITLE_ABS:("dose-volume histogram" OR "dosimetric parameter*" OR "dose-volume"
OR "normal tissue complication probability" OR "NTCP" OR "organ* at risk"
OR "OAR" OR "dose constraint*" OR "rectal dose" OR "bladder dose"
OR "urethral dose" OR "bladder trigone" OR "anal sphincter" OR "anal canal")
NOT TITLE_ABS:(child* OR pediatric OR paediatric)
AND PUB_YEAR:[2000 TO 2026] AND LANG:"eng"
```

Text S1.3. Immunological/inflammatory biomarkers (Search ID S3).

PubMed/MEDLINE:

```
("Prostatic Neoplasms"[MeSH] OR "prostate cancer"[tiab] OR "prostatic neoplasm*"[tiab])
AND ("Radiotherapy"[MeSH] OR "Radiotherapy, Intensity-Modulated"[MeSH] OR "Radiosurgery"[MeSH]
OR "radiotherapy"[tiab] OR "radiation therapy"[tiab] OR "external beam radiotherapy"[tiab]
OR "EBRT"[tiab] OR "IMRT"[tiab] OR "intensity-modulated radiotherapy"[tiab] OR "VMAT"[tiab]
OR "volumetric modulated arc therapy"[tiab] OR "SBRT"[tiab] OR "stereotactic body
radiotherapy"[tiab])
AND ("Radiation Injuries"[MeSH] OR "genitourinary toxicity"[tiab] OR "urinary toxicity"[tiab]
OR "gastrointestinal toxicity"[tiab] OR "rectal toxicity"[tiab] OR "radiation-induced
toxicity"[tiab]
OR "radiotoxicity"[tiab])
AND ("cytokine*"[tiab] OR "inflammatory biomarker*"[tiab] OR "interleukin*"[tiab] OR "IL-
6"[tiab]
OR "interleukin-6"[tiab] OR "TGF-beta"[tiab] OR "transforming growth factor"[tiab]
OR "TNF-alpha"[tiab] OR "tumor necrosis factor"[tiab] OR "C-reactive protein"[tiab] OR
"CRP"[tiab]
OR "lymphocyte*"[tiab] OR "lymphopenia"[tiab] OR "systemic immune-inflammation index"[tiab]
OR "SII"[tiab] OR "macrophage colony-stimulating factor"[tiab] OR "M-CSF"[tiab] OR "CD4"[tiab]
OR "lymphocyte apoptosis"[tiab] OR "extracellular vesicle*"[tiab] OR "myeloperoxidase"[tiab])
NOT (child*[tiab] OR pediatric[tiab] OR paediatric[tiab])
AND ("2000"[PDAT] : "2026"[PDAT]) AND English[la]
```

Scopus:

```
TITLE-ABS-KEY(("prostatic neoplasms" OR "prostate cancer" OR "prostatic neoplasm*")
AND ("radiotherapy" OR "radiation therapy" OR "external beam radiotherapy" OR "EBRT"
OR "IMRT" OR "intensity-modulated radiotherapy" OR "VMAT" OR "volumetric modulated arc
therapy"
OR "SBRT" OR "stereotactic body radiotherapy"))
AND TITLE-ABS-KEY(("genitourinary toxicity" OR "urinary toxicity" OR "gastrointestinal
toxicity"
OR "rectal toxicity" OR "radiation-induced toxicity" OR "radiotoxicity")
AND ("cytokine*" OR "inflammatory biomarker*" OR "interleukin*" OR "IL-6"
OR "TGF-beta" OR "transforming growth factor" OR "TNF-alpha"
OR "tumor necrosis factor" OR "C-reactive protein" OR "CRP"
OR "lymphocyte*" OR "lymphopenia" OR "systemic immune-inflammation index"
OR "SII" OR "macrophage colony-stimulating factor" OR "M-CSF"
OR "CD4" OR "lymphocyte apoptosis" OR "extracellular vesicle*" OR "myeloperoxidase"))
AND NOT TITLE-ABS-KEY(child* OR pediatric OR paediatric)
AND PUBYEAR > 1999 AND PUBYEAR < 2027 AND LANGUAGE(english)
```

Europe PMC:

```
TITLE_ABS:("prostatic neoplasms" OR "prostate cancer" OR "prostatic neoplasm*")
AND TITLE_ABS:("radiotherapy" OR "radiation therapy" OR "external beam radiotherapy" OR "EBRT"
OR "IMRT" OR "intensity-modulated radiotherapy" OR "VMAT" OR "volumetric modulated arc
therapy"
OR "SBRT" OR "stereotactic body radiotherapy")
AND TITLE_ABS:("genitourinary toxicity" OR "urinary toxicity" OR "gastrointestinal toxicity"
OR "rectal toxicity" OR "radiation-induced toxicity" OR "radiotoxicity")
AND TITLE_ABS:("cytokine*" OR "inflammatory biomarker*" OR "interleukin*" OR "IL-6"
OR "TGF-beta" OR "transforming growth factor" OR "TNF-alpha"
OR "tumor necrosis factor" OR "C-reactive protein" OR "CRP"
OR "lymphocyte*" OR "lymphopenia" OR "systemic immune-inflammation index"
OR "SII" OR "macrophage colony-stimulating factor" OR "M-CSF"
OR "CD4" OR "lymphocyte apoptosis" OR "extracellular vesicle*" OR "myeloperoxidase")
NOT TITLE_ABS:(child* OR pediatric OR paediatric)
AND PUB_YEAR:[2000 TO 2026] AND LANG:"eng"
```

Text S1.4. Radiogenomics/genetic factors (Search ID S4).

PubMed/MEDLINE:

```
("Prostatic Neoplasms"[MeSH] OR "prostate cancer"[tiab] OR "prostatic neoplasm*"[tiab])
AND ("Radiotherapy"[MeSH] OR "Radiotherapy, Intensity-Modulated"[MeSH] OR "Radiosurgery"[MeSH]
OR "radiotherapy"[tiab] OR "radiation therapy"[tiab] OR "external beam radiotherapy"[tiab]
OR "EBRT"[tiab] OR "IMRT"[tiab] OR "intensity-modulated radiotherapy"[tiab] OR "VMAT"[tiab]
OR "volumetric modulated arc therapy"[tiab] OR "SBRT"[tiab] OR "stereotactic body
radiotherapy"[tiab])
AND ("Radiation Injuries"[MeSH] OR "genitourinary toxicity"[tiab] OR "urinary toxicity"[tiab]
OR "gastrointestinal toxicity"[tiab] OR "rectal toxicity"[tiab] OR "radiation-induced
toxicity"[tiab]
OR "radiotoxicity"[tiab])
AND ("radiogenomic*"[tiab] OR "genetic polymorphism*"[tiab] OR "single nucleotide
polymorphism*"[tiab]
OR "SNP"[tiab] OR "microRNA"[tiab] OR "miRNA"[tiab] OR "genome-wide association"[tiab]
OR "GWAS"[tiab] OR "DNA methylation"[tiab] OR "PROSTOX"[tiab] OR "ATM"[tiab] OR "TGFB1"[tiab]
OR "RNASEL"[tiab] OR "polygenic risk score"[tiab] OR "candidate gene"[tiab]
OR "radiosensitivity"[tiab] OR "radiogenomics"[tiab])
NOT (child*[tiab] OR pediatric[tiab] OR paediatric[tiab])
AND ("2000"[PDAT] : "2026"[PDAT]) AND English[la]
```

Scopus:

```
TITLE-ABS-KEY(("prostatic neoplasms" OR "prostate cancer" OR "prostatic neoplasm*")
AND ("radiotherapy" OR "radiation therapy" OR "external beam radiotherapy" OR "EBRT"
OR "IMRT" OR "intensity-modulated radiotherapy" OR "VMAT" OR "volumetric modulated arc
therapy"
OR "SBRT" OR "stereotactic body radiotherapy"))
AND TITLE-ABS-KEY(("genitourinary toxicity" OR "urinary toxicity" OR "gastrointestinal
toxicity"
OR "rectal toxicity" OR "radiation-induced toxicity" OR "radiotoxicity")
AND ("radiogenomic*" OR "genetic polymorphism*" OR "single nucleotide polymorphism*"
OR "SNP" OR "microRNA" OR "miRNA" OR "genome-wide association"
OR "GWAS" OR "DNA methylation" OR "PROSTOX" OR "ATM" OR "TGFB1"
OR "RNASEL" OR "polygenic risk score" OR "candidate gene"
OR "radiosensitivity" OR "radiogenomics"))
AND NOT TITLE-ABS-KEY(child* OR pediatric OR paediatric)
AND PUBYEAR > 1999 AND PUBYEAR < 2027 AND LANGUAGE(english)
```

Europe PMC:

```
TITLE_ABS:("prostatic neoplasms" OR "prostate cancer" OR "prostatic neoplasm*")
AND TITLE_ABS:("radiotherapy" OR "radiation therapy" OR "external beam radiotherapy" OR "EBRT"
OR "IMRT" OR "intensity-modulated radiotherapy" OR "VMAT" OR "volumetric modulated arc
therapy"
OR "SBRT" OR "stereotactic body radiotherapy")
AND TITLE_ABS:("genitourinary toxicity" OR "urinary toxicity" OR "gastrointestinal toxicity"
OR "rectal toxicity" OR "radiation-induced toxicity" OR "radiotoxicity")
AND TITLE_ABS:("radiogenomic*" OR "genetic polymorphism*" OR "single nucleotide polymorphism*"
OR "SNP" OR "microRNA" OR "miRNA" OR "genome-wide association"
OR "GWAS" OR "DNA methylation" OR "PROSTOX" OR "ATM" OR "TGFB1"
OR "RNASEL" OR "polygenic risk score" OR "candidate gene"
OR "radiosensitivity" OR "radiogenomics")
NOT TITLE_ABS:(child* OR pediatric OR paediatric)
AND PUB_YEAR:[2000 TO 2026] AND LANG:"eng"
```
